# Identification of a Profile of Neutrophil-Derived Granule Proteins in the Surface of Gold Nanoparticles after Their Interaction with Human Breast Cancer Sera

**DOI:** 10.3390/nano10061223

**Published:** 2020-06-23

**Authors:** María del Pilar Chantada-Vázquez, María García-Vence, Sergio Vázquez-Estévez, Susana B. Bravo, Cristina Núñez

**Affiliations:** 1 Research Unit, Hospital Universitario Lucus Augusti (HULA), Servizo Galego de Saúde (SERGAS), 27002 Lugo, Spain; mariadelpilarchantadavazquez@gmail.com; 2 Proteomic Unit, Instituto de Investigaciones Sanitarias, Complejo Hospitalario Universitario de Santiago de Compostela (IDIS-CHUS), 15706 Santiago de Compostela, Spain; mariagarve@outlook.es; 3 Oncology Division, Hospital Universitario Lucus Augusti (HULA), Servizo Galego de Saúde (SERGAS), 27002 Lugo, Spain; Sergio.Vazquez.Estevez@sergas.es

**Keywords:** breast cancer (BC), gold nanoparticles (AuNPs), surface, protein biomarkers, neutrophils, mass spectrometry (MS)

## Abstract

It is well known that the interaction of a nanomaterial with a biological fluid leads to the formation of a protein corona (PC) surrounding the nanomaterial. Using standard blood analyses, alterations in protein patterns are difficult to detect. PC acts as a “nano-concentrator” of serum proteins with affinity for nanoparticles’ surface. Consequently, characterization of PC could allow detection of otherwise undetectable changes in protein concentration at an early stage of a disease, such as breast cancer (BC). Here, we employed gold nanoparticles (AuNPs_diameter_: 10.02 ± 0.91 nm) as an enrichment platform to analyze the human serum proteome of BC patients (n = 42) and healthy controls (n = 42). Importantly, the analysis of the PC formed around AuNPs after their interaction with serum samples of BC patients showed a profile of proteins that could differentiate breast cancer patients from healthy controls. These proteins developed a significant role in the immune and/or innate immune system, some of them being neutrophil-derived granule proteins. The analysis of the PC also revealed serum proteome alterations at the subtype level.

## 1. Introduction

Breast cancer (BC) rises to 1.4 million new cases worldwide each year [1]. Regarding BC treatment, the clinical decision depends on the identification of prognostic factors revealed from classical pathophysiological and clinical data (tumor size, grade, and presence or absence of positive lymph nodes) [2].

BC is currently classified into five intrinsic subtypes, that have been defined as follows: (1) luminal A subtype or LA (estrogen receptor (ER) positive, human epidermal growth factor receptor 2 (HER2) negative, Ki-67 cell proliferation marker low, and progesterone receptor (PR) high); (2) luminal B-HER2 negative or LB− (ER positive, HER2 negative, and either Ki-67 high or PR low); (3) luminal B-HER2 positive or LB+ (ER positive, HER2 overexpressed or amplified, any Ki-67, and any PR); (4) HER2 positive or HER2+ (HER2 over-expressed or amplified, ER and PR absent); and (5) triple negative breast cancer or TNBC (ER and PR absent and HER2 negative) [3]. In the clinical practice, expression levels of these biomarkers are normally used to classify patients for prognostic predictions and for the selection of a good treatment option [4,5,6,7]. 

Proteins that could improve current BC classifications could be detected using novel proteomics tools [8,9,10]. Moreover, proteomics might identify protein biomarkers defining differences in prognosis, therapy resistance and metastatic spread within a specific subtype.

Some proteins and peptides have been identified as breast cancer biomarkers in nipple aspirate fluid [11], breast tumor tissue [12], and serum [13]. Notably, serum biomarkers present the advantage that the sample is obtained through a minimally invasive method and can yield valuable information about the breast cancer status. Furthermore, serum biomarkers represent a source of potential markers for the prognosis or diagnosis of breast cancer, and also could be potential drug targets.

However, the discovery and validation of protein biomarkers presented in serum are masked by the presence of high-molecular-weight (HMW) proteins, such as immunoglobulins and serum albumin, which comprise 90% of the proteins present in serum. A high abundance of HMW proteins hides the low-molecular-weight (LMW) proteins when employing standard protein detection methods [14]. To overcome this limitation, different methods have been reported to remove or minimize the presence of such abundant proteins, such as on-chip automated platforms, commercial kits, immunoaffinity chromatographic columns containing immobilized antibodies, and chemical reagents (acetonitrile and dithiothreitol) [15]. 

Due to candidate biomarkers comprising less than 1% of serum proteins and being present in very low concentrations, it is necessary to isolate and enrich LMW proteins from complex mixtures for biomarker discovery. Different methods for the enrichment of LMW proteins from biofluids to find disease-associated biomarkers were reported [16]. 

In this way, different nanoparticles emerged as promising sorbent materials used in the enrichment of low-abundance peptides/proteins for subsequent mass spectrometric identification [17]. 

It is well known that the interaction of a nanomaterial with a biological fluid leads to the formation of a protein corona (PC) surrounding the nanomaterial. Using standard blood analyses, alterations in protein patterns are difficult to detect. Importantly, PC acts as a “nano-concentrator” of serum proteins with affinity for nanoparticles’ surface. Consequently, the characterization of PC could allow for the detection of otherwise undetectable changes in protein concentration at an early stage of a disease, or after chemotherapy or surgery [18]. 

The protein composition and content in the corona depend on several parameters, such as: (i) physicochemical properties of the NPs (i.e., size, composition, curvature, shape, surface charge and surface chemistry, hydrophobicity/hydrophilicity) [19,20,21]; (ii) characteristics of the biological media (i.e., temperature, and protein source) [22,23,24]; (iii) incubation time [25]. 

Our team applied different nanoparticles for the pre-fractionation/enrichment of proteins/peptides present in human serum [26,27,28] to discovery novel blood-based protein biomarkers of BC [29]. It was also found that the interaction of a nanomaterial with a biological fluid leads to the formation of a protein corona (PC) surrounding the nanomaterial, which is strongly influenced by the patient’s specific disease, named personalized protein corona (PPC) [30]. Applying this new concept, C. Núñez and co-workers found potential biomarkers for the prognosis and follow-up of triple negative breast cancer (TNBC) patients [31]. 

In the present study, 42 healthy women (named healthy controls (HC)) and 42 BC patients categorized into 5 subtypes, namely luminal A (n = 11), luminal B-HER2 negative (n = 10), luminal B-HER2 positive (n = 7), HER2 positive (n = 6), and triple negative (n = 8) were recruited. 

Due to the colloidal stability, the high surface-area-to-volume ratio and the ability to conjugate with biomolecules [32], gold nanoparticles (AuNPs: 10.02 ± 0.91 nm) were used to incubate with serum samples of breast cancer patients and healthy controls, and the resultant protein coronas were thoroughly characterized and compared by mass spectrometry (MS)-based proteomics.

## 2. Materials and Methods 

### 2.1. Reagents

All reagents used were HPLC grade or electrophoresis grade. Acrylamide/bis-acrylamide 30% solution (37.5:1), ammonium bicarbonate (ambic), *β*-mercaptoethanol, Coomassie Brilliant Blue R250 (CBB), dithiothreitol (DTT), iodoacetamide (IAA), formic acid, glycerol 86–88%, sodium borohydride (NaBH_4_), sodium carbonate, sodium citrate tribasic dihydrate (HOC(COONa)(CH_2_COONa)_2_·2H_2_O), (*N,N,N*, *N*′-tetramethylethylenediamine (TMED), trifluoroacetic acid, tris-base, trypsin, and the Sigma Marker wide range 6.5–200 KDa were all from Merck (Barcelona, Spain). Acetonitrile, formaldehyde, methanol and sodium dodecyl sulfate (SDS) were supplied by *Panreac Química SLU* (Barcelona, Spain). Bromophenol-blue was purchased from Riedel-de Haen (Seelze, Germany). Pierce™ Trypsin Protease, MS Grade was purchased from Thermo Fisher Scientific (Bremen, Spain). Hydrogen tetrachloroaurate (III) hydrate (HAuCl_4_·xH_2_O) (99.9% Au) (49% Au) at 10% *w*/*v* was acquired from Strem Chemicals (Kehl, Germany). 

### 2.2. Apparatus

Transmission electron microscopy (TEM) images of AuNPs were captured by a transmission electron microscope (Jeol JEM 1011 microscope) from CACTUS, University of Santiago de Compostela, Spain. For the preparation of the samples before the TEM determination, a drop of the gold colloidal dispersion was placed onto an ultrathin carbon-coated copper grid, and after that, the solvent was evaporated. The transmission electron microscopy (TEM) method provided two-dimensional images of nanoparticles which were used to produce number-based size distributions and calculate the diameter of nanoparticles.

Zeta potential (ζ) measurements of AuNPs were performed on a Malvern Zetasizer Nano ZS instrument from University of Santiago de Compostela (Spain) at 25 °C, realizing 3 determinations per sample. Protein separation by sodium dodecyl sulfate-polyacrylamide gel electrophoresis (SDS-PAGE) was developed in a Power Pac Basic power supply from Bio-Rad (CA, USA). A Qubit™ 4 Quantitation Starter Kit from Thermo Fisher Scientific (Bremen, Spain) was used for the protein quantification by measuring the absorbance at 280 nm (Abs_280 nm_). 

### 2.3. Synthesis of Citrate-Gold Nanoparticles (AuNPs)

AuNPs were synthesized in aqueous solution following the citrate reduction method [31,33]. Briefly, a 10% *w*/*v* of HAuCl_4_·xH_2_O (54 μL) was slowly added to a stirred solution of 60 mL of a HOC(COONa)(CH_2_COONa)_2_·2H_2_O (0.075% *w*/*v*) at 100 °C. The resulting reaction mixture was kept under reflux until the color turned from yellow to deep red, showing the formation of the colloidal dispersion. The solution of AuNPs was cooled (at room temperature) and stored at 4 °C (maximum one month).

### 2.4. Sample Resources

Blood samples were collected from newly diagnosed BC patients at Hospital Universitario Lucus Augusti of Lugo, Spain. Participating patients do not have received any type of preoperative radio or chemotherapy, and not present a history of any noteworthy systemic diseases.

Peripheral venous blood samples were collected in VACUETTE^®^ serum clot activator tubes (Kremsmünster, Austria) (10 mL) before the breast patients underwent surgery and/or before receiving any systemic treatment (chemotherapy, hormone therapy, anti-HER2 therapy), and radiotherapy. The exclusion criteria for the breast cancer patients was: (a) present a history of some type of mammary pathology; (b) diagnosis and/or treatment of other types of cancer (with the exception of basal or squamous cell skin cancers properly treated or resected cervix cancer); (c) chronic disease under medical treatment; (d) clinical history of intestinal problems and/or malabsorption (Crohn′s disease, irritable bowel syndrome (IBS), colitis, etc.); and (e) previous or current positive test of infectious disease potentially transmissible during the manipulation of the biological samples (HIV, hepatitis, tuberculosis, etc.).

From June 2017 to June 2018, a total of 42 blood samples (age varying from 26 to 81 years) from BC patients were collected. Histopathological reports were used to corroborate Her2 neu receptor and hormonal receptor status. Table 1 summarizes overall clinical features of BC patients, including histology, clinical stage, tumor size, receptor status, and nodal status.

In this work, breast cancer patients (BC) (n = 42) were categorized into five subtypes: luminal A subtype or LA (n = 11), luminal B-HER2 negative or LB− (n = 10), luminal B-HER2 positive or LB+ (n = 7), HER2 positive or HER2+ (n = 6), and triple-negative breast cancer or TNBC (n = 8). 

Age-matched healthy controls (HC) (n = 42) selected were devoid of diabetes or hypertension and any hormonal or medical supplementation administered during the last 3 months. Peripheral venous blood samples were also collected in VACUETTE® Serum Clot Activator Tubes (10 mL).

This study was approved by the Clinical Research Ethics Committees (CEIC) of Galicia, Spain. Informed consent forms were ethically obtained from all women (healthy controls and BC patients) before sample collection.

### 2.5. Depletion, Reduction and Alkylation of Proteins Presented in Human Serum Samples (Healthy Controls and BC Patients)

Blood samples were centrifuged at 1800× *g* (5 min, 4 °C), and serum aliquots were stored at −80 °C, until their analysis.

Before the interaction of the proteins presented in human serum with AuNPs (10.02 ± 0.91 nm), the depletion of multiple high abundant proteins was carried out with dithiothreitol (DTT) following the method reported by Warder el al. [34,35]. In brief, human serum (n = 2) were filtered with Miller-GP^®^ Filter Units (Millipore, Bedford, MA, USA) with a size of 0.22 μm. Filtered serum samples (30 μL) were mixed with fresh DTT 500 mM (3.3 μL) in milli-Q and vortex 30 s. After the incubation at room temperature (*ca.* 60 min), the viscous white precipitate formed was eliminated by centrifugation at 18,840× *g* (20 min). Supernatants were collected to new tubes. Another function of DTT was the reduction of disulfide bonds, thus proteins presented in supernatants are reduced. The resulting cysteines were then alkylated with iodoacetic acid (IAA) 400 mM (μL) under incubation room temperature in the dark (45 min).

### 2.6. Interaction of Proteins Presented in Human Serum Samples with the Surface of AuNPs: Formation of the Protein Corona (PC)

After the depletion of multiple high abundant proteins presented in all human serum samples, and the reduction/alkylation of the remaining proteins, the interaction of the latter with AuNPs (10.02 ± 0.91 nm) was carried out following a previously reported method [31,33]. Briefly, 75 μL of AuNPs and 40 μL of a citrate/citric acid buffer were added to each different serum samples, adjusting the pH to 5.8. Then, AuNPs-serum solutions were incubated with shaking in a thermostatic bath at 37 °C for 30 min. The nanoparticles with serum bound proteins (PC) were harvested by centrifugation at 18,840× *g* (30 min). The pellet formed was washed with 25 μL citrate/citric acid buffer and harvested again (×3) by centrifugation at 18,840× *g* (30 min) to remove serum proteins unbound to the AuNPs surface.

### 2.7. Separation of Serum Proteins Bound to the AuNPs Surface by 1-D gel Electrophoresis and Identification by Mass Spectrometry (LC-MS/MS)

Once pellets and supernatants were resuspended in loading buffers following the method described in previous work [31,33], they were vortexed (1 min) and denatured by heating at 100 °C (5 min). Then 5 μL of the samples were loaded on the SDS-PAGE gel (1 mm thickness) and proteins separated at 180 V (constant voltage) (120 min). After electrophoresis, the gels were stained with Colloidal Coomassie Blue (CBB) [36]. After incubation, gels were destained [31,33].

Protein bands were manually excised from the gels, and the protein digestion with trypsin was carried out following a previously reported method [37]. Digested peptides were separated by reverse-phase chromatography (RPC), and identification of proteins was revealed with a nanoLC 400 system (Eksigent Tech., Dublin, CA, USA) coupled to the Triple TOF 6600 mass spectrometer (AB Sciex, Toronto, ON, CA). Proteins were searched with the ProteinPilot^TM^ software (version 5.0.1; AB Sciex) through the human-specific Uniprot database with 1 missed cleavage and 1% global false discovery rate (FDR) as input parameters [38,39].

### 2.8. Protein Functional Interaction Network Analysis and Protein Ontology Classification

The functional interaction networks of the proteins were processed using the Search Tool for the Retrieval of Interacting Genes/Proteins (STRING v.10.0 database; http://string-db.org) [40]. The STRING database offers integration and analysis of indirect and direct (physical) protein–protein interactions (PPI) as well as coverages on the functional association. The list of protein names was inserted into a table to deliver the network of protein–protein interactions. Functionally associated proteins frequently have analogous phylogenetic profiles and/or exhibit the phenomenon of co-expression. 

The PANTHER (Protein Analysis Through Evolutionary Relationships) classification system (http://www.pantherdb.org/) was the tool used for the protein ontology classification. The differentially expressed proteins in the BC patients were grouped according to their protein classes.

## 3. Results and Discussion

Blood samples of 42 BC patients and 42 HC were collected. The group of BC patients was divided into the different subtypes: LA (n = 11), LB− (n = 10), LB+ (n = 7), HER2+ (n = 6), and TNBC (n = 8). All samples have been recruited, processed and analyzed in the same way. 

The treatment of the serum samples with DTT resulted in the elimination of the high abundant proteins and the reduction of the disulfide bonds of proteins presented in the remaining fraction. The resulting cysteines were then alkylated (blocked) with IAA. 

Gold nanoparticles (AuNPs) exhibit colloidal stability, high surface-area-to-volume ratio and the ability to conjugate with biomolecules. For this reason, AuNPs emerged as promising sorbent materials used in the enrichment of low-abundance peptides/proteins for subsequent mass spectrometric identification. In the present work, we used AuNPs (10.02 ± 0.91 nm) to pre-concentrate low-abundance proteins presented in the sera of BC patients (n = 42) and healthy controls (n = 42), and the resultant protein coronas were thoroughly characterized to identify potential protein biomarkers.

The formation of the PC is a dynamic process that depends on multiple factors, including the physicochemical properties of the nanomaterials such as size, composition, curvature shape, surface chemistry and surface charge, hydrophobicity/hydrophilicity. Furthermore, other parameters that also influenced the formation of the PC are the characteristics of biological media, such as protein concentrations, protein source, and choice of anticoagulant and flow status. 

Importantly, the pH, temperature, and incubation time are factors that also modulate the interaction of NPs with biological media. In the present work, the interaction of colloidal AuNPs (10.02 ± 0.91 nm) with serum samples was carried out adjusting the pH to 5.8, at 37 °C for 30 min, following an optimized protocol [31,33]. Thus, serum samples were further processed in duplicate as described in Section 2.6. 

TEM and ζ-potential measurements showed that after the incubation of AuNPs (10.02 ± 0.91 nm) with the serum of HC and BC patients, an increase in their size was observed due to the formation of the PC, from 10.02 ± 0.91 nm to 12.17 ± 0.98 nm and 12.14 ± 0.82 nm, respectively. After the incubation with serum, the mean particle surface charge of the AuNPs (10.02 ± 0.91 nm) became less negative ranging from −37.0 mV to −29.7 mV. This effect is probably due to a preferential interaction between the AuNPs surface with positively charged proteins [41,42] (see Figures S1–S3).

After the interaction of reduced and alkylated serum aliquots with AuNPs (10.02 ± 0.91 nm), two protein fractions were obtained in each case: (a) a pellet fraction of proteins bound to the nanoparticle surface, named protein corona (PC), and (b) a supernatant of unbound proteins that was discarded. Only the proteins of the pellet fraction (PC) were separated by 1D-SDS-PAGE, digested with trypsin and identified by mass spectrometry (LC-MS/MS). 

### 3.1. Proteins Identified in the AuNP–protein Corona by Shotgun Proteomics Techniques 

Once all samples were processed, all proteins presented in the protein corona were analyzed, resulting in a large number of proteins identified.

**Figure 1:**
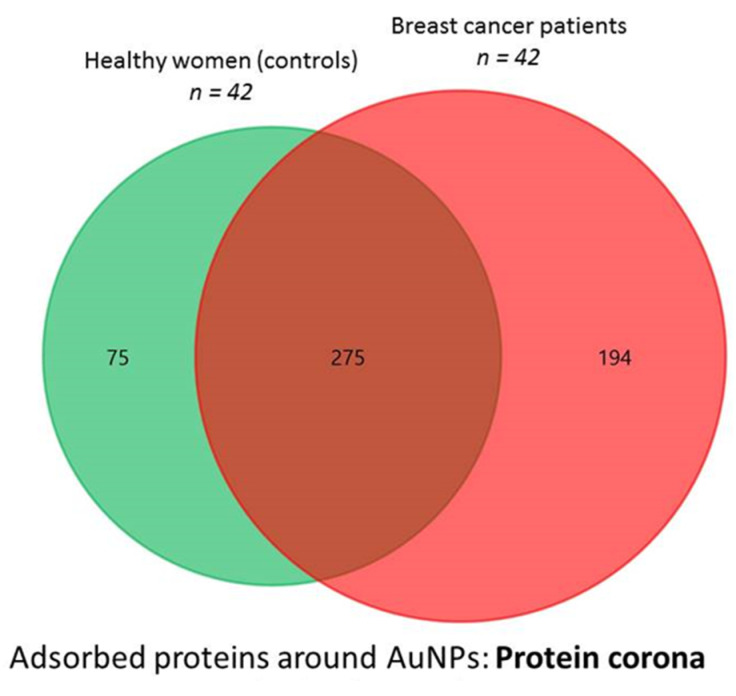
Venn diagram showing the number of proteins identified in the AuNP–protein corona formed after the interaction of AuNPs (10.02 ± 0.91 nm) with serum samples belonging to 42 healthy women (controls) and 42 breast cancer patients.

A total of 350 and 469 proteins were commonly identified in the surface of AuNPs (10.02 ± 0.91 nm) after their incubation with all serum samples (n = 2) belonging to 42 HC and 42 BC patients, respectively. From them, 275 were commonly found in both sample groups (see Figure 1). Thus, fractionation of the proteome using AuNPs (10.02 ± 0.91 nm), allowed the identification of 194 proteins as potential biomarkers of BC (see Table S1), of which 72 proteins have been found in patients with the LA subtype, 82 in the LB−, 59 in the LB+, 36 in the HER2+ and 59 in the TNBC subtype (see Tables S1–S3). 

The search of differential proteins (biomarkers) between the BC subtypes is very important to know how this disease varies according to its classification and to know different ways in which these proteins are involved. Thus, a comparison between the proteins identified among the different BC subtypes was also shown.

**Figure 2:**
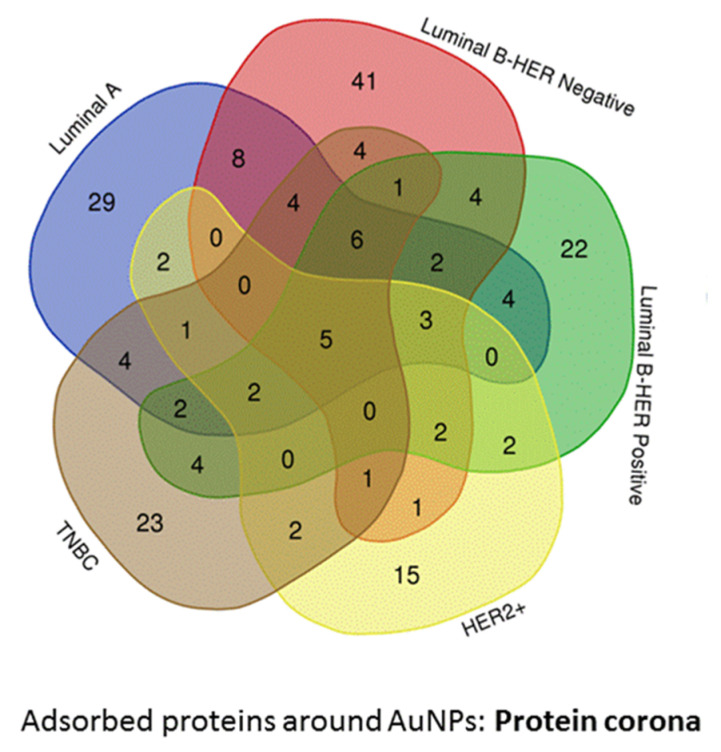
Venn diagram representing the number of shared proteins (biomarkers) identified in the AuNPs-protein corona formed after the interaction of AuNP (10.02 ± 0.91 nm) with serum samples belonging to the different breast cancer subtypes: luminal A (n = 11), luminal B HER2 negative (n = 10), luminal B HER2 positive (n = 7), HER2 positive (n = 6), and triple negative (n = 8).

Figure 2 showed the distribution of the 194 proteins (biomarkers) identified in the AuNPs-protein corona in the different BC subtypes. Particularly, 29, 41, 22, 15 and 23 are unique proteins of the BC subtypes LA, LB−, LB+, HER2+, and TNBC, respectively (see Table 2 and Table S3). 

Five proteins were commonly found in the AuNPs-protein corona for all BC patients, regardless the BC subtype: properdin (CFP), immunoglobulin heavy variable 1-2 (IGHV1-2), phosphatidylcholine-sterol acyltransferase (LCAT), cadherin-5 (CDH5) and actin, cytoplasmic 2 (ACTG1). From them, recent glycoproteomic studies have found that CDH5 levels and CDH5 glycosylation are potential serological biomarkers to distinguish BC patients metastatic from those metastasis-free [43,44].

### 3.2. The Biological Role of the Proteins Identified in the AuNP–Protein Corona

The protein interaction network diagram was constructed for the 194 serum differential proteins identified in the AuNP–protein corona after the interaction of AuNPs (10.02 ± 0.91 nm) (30 min, incubation) with serum samples belonging to 42 BC patients with different subtypes. The interactions of proteins codified by the 194 selected genes were explored by the use of the STRING online tool.

A total of 232 protein–protein interactions were shown, whereas the estimated was 129 in the network analysis (see Figure S4), with an average local clustering coefficient of 0.336. This indicates that the set of proteins codified by the 194 initially selected genes presented more interactions between themselves than that expected for a random set of proteins of similar size, drawn from the genome. Such enhancement reveals that these proteins are linked as a biologic group.

**Figure 3:**
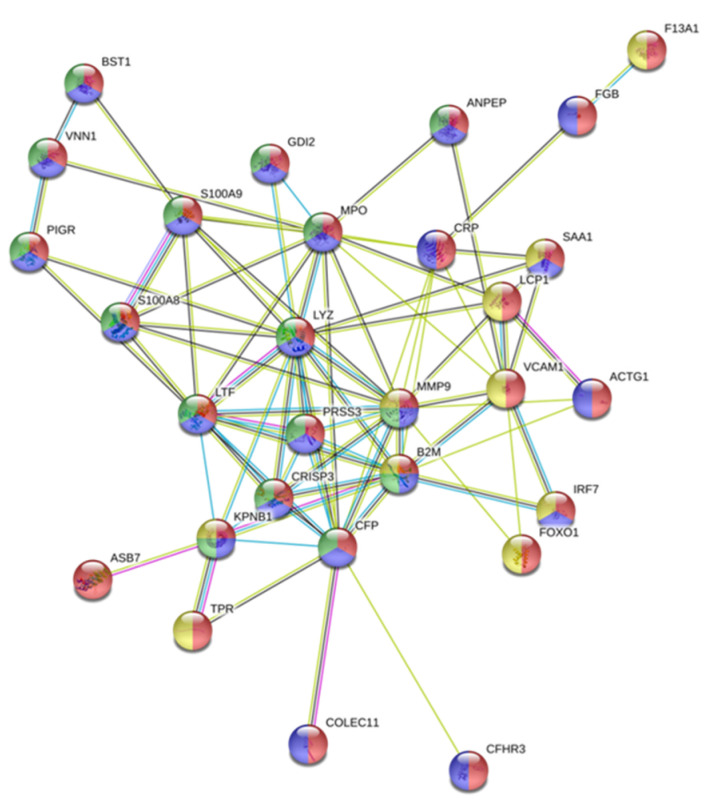
The cluster found in the protein–protein interaction network map of the 194 genes encoded differentially proteins identified in the AuNP–protein corona after the interaction of AuNPs (10.02 ± 0.91 nm) (30 min, incubation) with serum samples belonging to 42 breast cancer patients. Based on the STRING database, 29 differential expressed proteins formed a network core with proteins of the immune system, of which some of them are neutrophil-derived granule proteins. The meaning of the different edge colors is related to the reactome pathways. Red: immune system; blue: innate immune system; yellow: cytokine signaling in the immune system, green: neutrophil degranulation.

In this analysis, 29 tumor-related proteins were located in the central area of the network as a core (see Figure 3). Importantly, all these proteins played a crucial role in the immune and/or innate immune system (F13A1, FGB, CRP, SAA1, LCP1, VCAM1, ACTG1, IRF7, FOXO1, ASB7, TPR, COLEC11, CFHR3), and some of them are neutrophil-derived granule proteins (BST1, VNN1, PIGR, GDI2, S100A8, S100A9, ANPEP, MPO, LYZ, LTF, PRSS3, CRISP3, KPNB1, MMP9, B2M, CFP) (see Figure 3).

Several proteins that are components of neutrophil granules have been related to cancer progression and they may be promising candidate therapeutic targets in states of tumor development and/or chronic inflammation [45]. Upon cell activation, these proteins are exposed at the cell surface or released to the extracellular medium [46].

Cytoplasmic granules of neutrophils are divided into three categories: (a) primary or azurophilic granules; (b) secondary or specific granules; and (c) tertiary or gelatinase granules [47]. While myeloperoxidase (MPO) is stored in the azurophil (primary) granules, proteins that play important roles in the interaction and degradation of the extracellular matrix (ECM) are contained in the specific (secondary) granules and tertiary granules, as matrix metalloproteinase-9 (MMP-9/gelatinase B) [48].

As previously mentioned, both neutrophil-derived granule proteins, MPO and MMP-9, were identified in the proteomic profile of some BC patients of the present study.

It was found that the extravasated neutrophils are responsible for the majority of the MMP-9 released in the tumor microenvironment, which induces angiogenesis via the release of VEGF from the ECM in many types of tumors, such as BC [49]. Furthermore, in breast secretions, as well as breast tissue with and without cancer, elevated levels of MPO-containing neutrophils were found, which are responsible for mammary tumor growth and enhanced metastases [50,51,52]. Thus, inhibition of MPO may limit tumor growth and reduce the rate of metastasis [53].

Another neutrophil-derived granule protein, properdin (CFP), was identified in all BC subtypes in the present study. Neutrophils can actively stabilize and intensify the alternative activation pathway of complement by secretion of CFP as part of the innate defense to produce an immune response [54].

As mentioned above, inflammation is the hallmark of the immune system activation, which develops a complex and specific role. Neutrophils are the first cells to appear on stage, whose secretory products amplify and modulate the inflammatory reaction [55]. Some of these products are pantetheinase (VNN1) [56], beta-2-microglobulin (B2M) [57] and the immunogenic protein calprotectin (S100A8/A9) [58]. Particularly, increased production and release of B2M are present in certain malignancies, including solid tumors like BC [59]. Furthermore, the level of B2M also is one of the most important independent prognostic factors and predictors of survival in patients with BC [60], and B2M played a role in creating multidrug-resistance [61].

The expression and function of S100A8 and S100A9 in BC is controversial. While some evidence supports a pro-malignancy role for S100A8 and S100A9 in breast cancer [62,63], other researchers consider that S100A8 and S100A9 expression is positive to BC prognosis [64,65]. In the present work, S100A8/A9 was identified in the serum of BC patients with the subtype LB+. Particularly, strong expression and secretion of S100A8/A9 were found to be implicated in the poor prognosis of Her2+/basal-like subtypes of BC [66].

Lactotransferrin (LTF) is also produced in neutrophils and stored in specific granules. Being a hormone-responsive gene, LTF may contribute to various hormone-dependent cancers, such as BC. F. Chekhun et al. [67] found a strong correlation between expression indexes of LTF and estrogen receptors (ER). Particularly, significantly higher expression of LF was found in ER-positive tumors than in ER-negative tumors (35 vs. 18%). These observations are in agreement with the results obtained in the present work because LTF was identified in the serum of BC patients with the LA subtype and TNBC patients.

The anticancer activity of LTF has been observed in different cancer cell lines, including BC cells [68]. In this context, lysozyme C (LYZ), a cornerstone of innate immunity, also acts as an anti-proliferative protein against different human cancer cells, such as BC cells [69]. Particularly, in the present work, LYZ was identified in the serum of BC patients with the LA subtype, as cysteine-rich secretory protein 3 (CRISP-3) that develops a role in innate immune defense [70]. Recently, lower expression of CRISP3 was associated with an improved DFS (disease-free survival) and OS (overall survival) in patients with mammary carcinoma [71].

On the other hand, the polymeric immunoglobulin receptor (PIGR) is one of the first-line antibodies produced in response to infection. It was also identified the ability of pIgR overexpression to promote cell migration and cancer metastasis, for example, in BC [72]. In the present work, this protein was identified in the serum of BC patients with the LA, LB− and TNBC subtypes.

Two enzymes that were found in the serum of BC patients are trypsin-3 (PRSS3) and aminopeptidase N (APN). Both are proteins whose enzymatic activity is fundamental for stimulating cancer and endothelial cell migration and invasion [73,74]. Particularly, PRSS3 [75] and APN [76] levels were significantly higher in breast tissues than in benign tissues. Furthermore, it was also found that APN, in combination with other standard prognostic factors, can be used as a prognostic factor in the assessment of BC prognosis [77].

In the present work, the Rab GDP dissociation inhibitor beta (GDI2) and the importin subunit beta-1 (KPNB1) were identified in the serum of BC with the LB− subtype. Recent studies revealed that GDI2 [78,79] and KPNB1 [80] proteins have a key role in the development of multiple tumors by controlling tumor progression, including that of BC [81,82]; thus, GDI2 and KPNB1could be valuable anticancer therapeutic targets [83].

**Figure 4:**
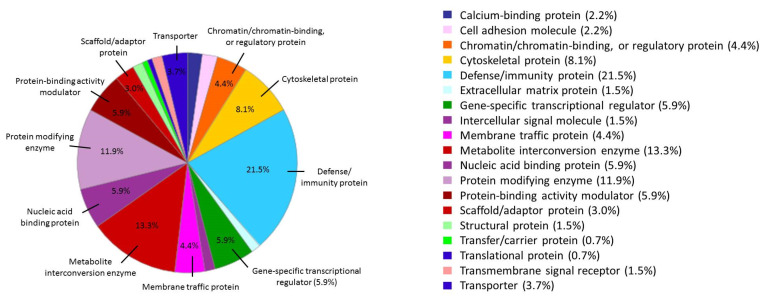
Functional classifications of the 194 specific proteins found in the AuNP–protein corona after the interaction of AuNPs (10.02 ± 0.91 nm) (30 min, incubation) with serum samples belonging to 42 breast cancer patients, according to their protein classes by PANTHER.

Furthermore, the 194 differential proteins found in the AuNPs-protein corona for the BC group, were classified according to their protein classes using the PANTHER database. Most of the differential proteins belonged to defense/immunity (21.5%), metabolite interconversion enzyme (13.3%), protein modifying enzyme (11.9%), cytoskeletal protein (8.1%), and gene-specific transcriptional regulator (5.9%). More classes of the differential proteins were also shown in Figure 4. Most of these 194 proteins played crucial roles in the immune system, probably due to the induction of an immune response in the microenvironment promoted by the breast tumor [84].

#### Molecular Function and Pathway Analysis for Subtype Specific Breast Cancer

**Figure 5:**
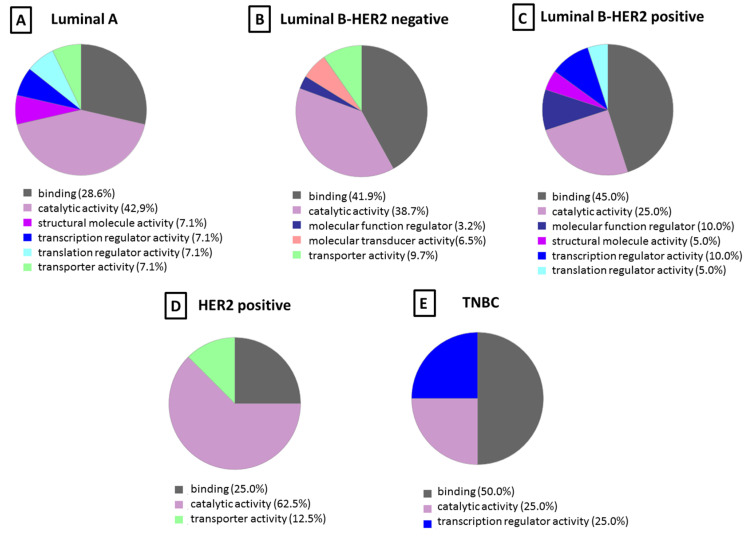
Classification according to the molecular function of the proteins identified in the protein corona from each breast cancer subtype: (**A**) luminal A, (**B**) luminal B-HER2 negative, (**C**) luminal B-HER2 positive, (**D**) HER2 positive (n = 6), and (**E**) triple negative. The percentage in each category is the ratio between the number of proteins in each category, indicated in parentheses, and total proteins identified in that fraction.

**Figure 6:**
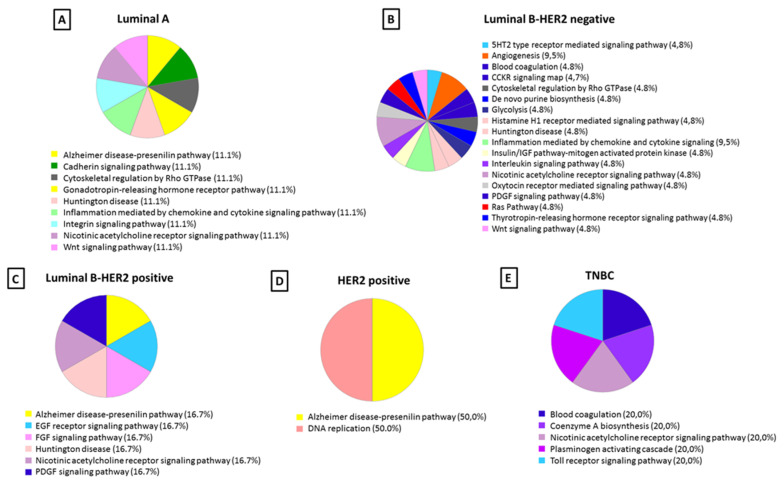
Classification according to the biological pathway of the proteins identified in the protein corona from each breast cancer subtype: (**A**) luminal A, (**B**) luminal B-HER2 negative, (**C**) luminal B-HER2 positive, (**D**) HER2 positive (n = 6), and (**E**) triple negative. The percentage in each category is the ratio between the number of proteins in each category, indicated in parentheses, and total proteins identified in that fraction.

The differentially identified proteins specific to each of the five subtypes of breast cancer found in the protein corona were subjected to PANTHER analysis to understand the molecular function (see Figure 5) and pathways (see Figure 6) altered in each case. 

The molecular functions most represented in the protein corona for all BC subtypes were catalytic (52.5–25.0%) and binding (50.0–25.0%) activities. While transporter activity (12.5–7.1%) was represented in the LA, LB− and HER2+ subtypes, structural (7.1–5.0%) was only represented LA and HER2+, with the remaining categories being minority ones (Figure 5).

The blood coagulation pathway was found to be altered in LB− and TNBC subtypes but not in LA, LB+, nor HER2+. After analysis of the protein corona, LB− and TNBC subtypes were found to be enriched in blood coagulation pathway with coagulation factor VII (FA7) and fibrinogen beta chain (FIBB), respectively.

Inflammation mediated by chemokine and cytokine signaling pathways was also seen to be an altered pathway in breast cancer, which was enriched only for the LA and LB− subtypes in the present study. Analysis of the protein corona showed that beta-actin-like protein 2 (ACTBL) was involved in this pathway in the LA subtype, while myosin-15 (MYH15) and 1-phosphatidylinositol 4,5-bisphosphate phosphodiesterase eta-1 (PLCH1) were involved in the LB− subtype.

Interestingly, the angiogenesis pathway was only observed in the LB− subtype, and ephrin type-B receptor (EPHB3) and coagulation factor VII (FA7) were the proteins involved in this pathway.

The epidermal growth factor (EGF) receptor signaling pathway is one of the most important pathways that regulate growth, survival, proliferation, and differentiation in mammalian cells [85]. Furthermore, it was also observed that cancer signaling pathways like epidermal growth factor (EGF) receptor and fibroblast growth factor (FGF) were majorly associated with HER2+. The protein SHC-transforming protein 3 (SHC3) was identified as the protein involved in both signaling pathways. In this way, SHC3 as a molecule implicated in both EGF receptor and FGF signaling pathways represented a potential activable target. Innovative anti-cancer drugs could be developed for the treatment of breast cancer patients with the HER2+ subtype based on the SHC3 target [86].

## 4. Conclusions

In this study, AuNPs (10.02 ± 0.91 nm) were shown to be a potential tool for the enrichment of disease-specific proteins of breast cancer in serum samples. The analysis of the PC formed around AuNPs (10.02 ± 0.91 nm) after their interaction with serum samples of 42 BC patients allowed the identification of 194 potential protein biomarkers, of which 72 have been found in patients with the LA subtype, 82 in the LB−, 59 in the LB+, 36 in the HER2+ and 59 in the TNBC subtype. The crucial role of these proteins in the immune system is probably due to the induction of an immune response in the microenvironment promoted by the breast tumor. Importantly, some of them are neutrophil-derived granule proteins that could be attracting candidate therapeutic targets in states of chronic inflammation and/or breast tumor development.

Further study to gain greater insight into the role of the immune system developed by the biomarkers found in the present study may expand novel therapeutic strategies for BC.

## Figures and Tables

**Table 1:** Clinical features of breast cancer tumors.

Characteristics/Patients		Number
Age (years)	<40	4
	40–59	21
	60–80	16
	>80	1
Tumor size (cm)	<2	25
	2–5	14
	>5	3
Histological types	In situ ductal carcinoma	2
	Invasive ductal carcinoma	36
	In situ lobular carcinoma	1
	Invasive lobular carcinoma	3
Receptor status	Luminal A	11
	Luminal B-HER2 negative	10
	Luminal B-HER2 positive	7
	HER2 positive	6
	Triple negative	8
Clinical stage	I	15
	II	20
	III	7
Nodal status	N0	25
	N1	17

**Clinical stages:** Stage I is characterized by either of these conditions: (A) the tumor is small, invasive, and has not spread to the lymph nodes; B) Cancer has spread to the lymph nodes and cancer in the lymph node is larger than 0.2 mm but less than 2 mm in size. There is either no evidence of a tumor in the breast or the tumor in the breast is 20 mm or smaller. Stage II is characterized by the following conditions: A) There is no evidence of a tumor in the breast, but cancer has spread to 1 to 3 axillary lymph nodes. It has not spread to distant parts of the body. (B) The tumor is 20 mm or smaller and has spread to the axillary lymph nodes. (C) The tumor is larger than 20 mm but not larger than 50 mm and has not spread to the axillary lymph nodes. (D) The tumor is larger than 20 mm but not larger than 50 mm and has spread to 1 to 3 axillary lymph nodes. E) The tumor is larger than 50 mm but has not spread to the axillary lymph nodes. Stage III is characterized by the following conditions: (A) The cancer of any size has spread to 4 to 9 axillary lymph nodes or to internal mammary lymph nodes. It has not spread to other parts of the body. Stage IIIA may also be a tumor larger than 50 mm that has spread to 1 to 3 axillary lymph nodes. (B): The tumor has spread to the chest wall or caused swelling or ulceration of the breast or is diagnosed as inflammatory breast cancer. It may or may not have spread to up to 9 axillary or internal mammary lymph nodes. It has not spread to other parts of the body. (C) A tumor of any size that has spread to 10 or more axillary lymph nodes, the internal mammary lymph nodes, and/or the lymph nodes under the collarbone. It has not spread to other parts of the body. **Nodal status:** *N0*: No regional lymph node metastases. *N1*: Metastases to a movable ipsilateral level I, II axillary lymph node(s).

**Table 2:** Specific proteins for each different breast cancer subtypes (luminal A (n = 11), luminal B HER2 negative (n = 10), luminal B HER2 positive (n = 7), HER2 positive (n = 6), and triple negative) identified in the AuNPs-protein corona after the interaction of AuNP (10.02 ± 0.91 nm) (30 min, incubation) with serum samples belonging to BC patients.

LA (n = 29)		LB− (n = 41)		LB+ (n = 22)	HER2+ (n = 15)	TNBC (n = 23)
LYZ	CRISP3	CTAGE9	GAPDH	IGLV3-9	PNMA6A	FAM110A
CRP	NEFH	KRT6C	GDI2	TNS3	CFAP100	PLD5
FILIP1L	SORBS1	IGHV7-4-1	MOAP1	ANPEP	TOP1	COG4
BST1	DCD	IGHV1-69D	AKAP9	IFT140	PRSS3P2	PTPRD
TFAP2E	KRT15	LRP2BP	FSIP2	TPR	OTOG	ZNF404
EXOC7	ALCAM	IGHV4-30-2	IGHV3-73	STXBP5L	ADAP2	SMC6
EIF3C	ADIPOQ	BRPF3	FGD6	SFTPB	SLC9A1	TRIM7
N/A	MPO	BLVRB	KRT31	SHC3	ZNF426	IGHV4-39
TTN	PHLDA1	HBD	KDM3B	TTC7A	MARK4	SUPT20H
WEE1	ABCB5	PLCH1	DES	MMP15	TSBP1	KMT2E
ACTBL2	LDHAL6A	AK6	CCDC28A	TBC1D1	SETD1A	PPCS
TACC2	KIF5B	IGHV3-53	EPHB3	EPAS1	MMP12	MYO15A
ANXA4	PGLS	AK1	CPD	GTPBP8	WFDC3	GRXCR2
NSUN6	N4BP1	HBG1	ELP3	IGHV3-20	SPATA9	SNX25
PNMA8C		PTPRG	CA3	CPEB4	ZGRF1	ZNF622
		SELENBP1	FFAR4	CCDC168		ASB7
		RPS6KA3	F7	DNAH3		KIF5A
		MYH15	MLLT1	PRDM5		IGHV3-21
		GSTO1	PRDX2	S100A9		FGB
		CPNE7	IGKV1-8	HHIPL2		MCF2L2
		KPNB1		S100A8		CCDC106
				LCP1		NRXN3
						IRF7

## Supplementary Materials

The following are available online at https://www.mdpi.com/2079-4991/10/6/1223/s1, Figure S1: TEM image of AuNPs@citrate in aqueous phase and the characterization data, Figure S2. TEM image of AuNPs@PC-controls in aqueous phase and the characterization data; Figure S3. TEM image of AuNPs@PC-BC in aqueous phase and the characterization data, Figure S4. Protein-protein interaction network map of the 194 genes encoded differentially proteins identified in the AuNP-protein corona after the interaction of AuNPs (10.02 ± 0.91 nm) (30 min, incubation) with serum samples belonging to 42 BC patients, Table S1. Number of proteins identified in the protein corona formed after the interaction of AuNPs (10.02 ± 0.91 nm) (30 min, incubation) with serum samples belonging to 42 healthy women (HC) and 42 breast cancer patients (BC) with different subtypes: luminal A (n = 11), luminal B HER2 negative (n = 10), luminal B HER2 positive (n = 7), HER2 positive (n = 6), and triple negative (n = 8); Table S2. Proteins identified in the protein corona formed after the interaction of AuNPs (10.02 ± 0.91 nm) (30 min, incubation) with serum samples belonging to 42 healthy women (HC) and 42 breast cancer patients (BC); Table S3. Proteins identified in the protein corona formed after the interaction of AuNPs (10.02 ± 0.91 nm) (30 min, incubation) with serum samples belonging to breast cancer patients with different subtypes: luminal A (n = 11), luminal B HER2 negative (n = 10), luminal B HER2 positive (n = 7), HER2 positive (n = 6), and triple negative (n = 8). The accession number, gene name and species (Human) were reported.

## References

[1] Siegel R.L., Miller K.D., Jemal A. (2017). Cancer statistics. CA Cancer J. Clin..

[2] Tong C.W.S., Wu M., Cho W.C.S., To K.K.W. (2018). Recent advances in the treatment of breast cancer. Front. Oncol..

[3] Inic Z., Zegarac M., Inic M., Markovic I., Kozomara Z., Djurisic I., Inic I., Pupic G., Jancic S. (2014). Difference between luminal A and luminal B subtypes according to Ki-67, tumor size, and progesterone receptor negativity providing prognostic information. Clin. Med. Insights Oncol..

[4] Perou C.M., Sørlie T., Eisen M.B., van de Rijn M., Jeffrey S.S., Rees C.A., Pollack J.R., Ross D.T., Johnsen H., Akslen L.A. (2000). Molecular portraits of human breast tumours. Nature.

[5] Goldhirsch A., Winer E.P., Coates A.S., Gelber R.D., Piccart-Gebhart M., Thürlimann B., Senn H.-J. (2013). Personalizing the treatment of women with early breast cancer: Highlights of the St Gallen International Expert Consensus on the Primary Therapy of Early Breast Cancer 2013. Ann. Oncol..

[6] Coates A.S., Winer E.P., Goldhirsch A., Gelber R.D., Gnant M., Piccart-Gebhart M., Thürlimann B., Senn H.-J. (2015). Tailoring therapies–improving the management of early breast cancer: St Gallen International Expert Consensus on the Primary Therapy of Early Breast Cancer 2015. Ann. Oncol..

[7] Sotiriou C., Neo S.Y., McShane L.M., Korn E.L., Long P.M., Jazaeri A., Martiat P., Fox S.B., Harris A.L., Liu E.T. (2003). Breast cancer classification and prognosis based on gene expression profiles from a population-based study. Proc. Natl. Acad. Sci. USA..

[8] Nakshatri H., Qi G., You J., Kerry B., Schneider B., Zon R., Buck C., Regnier F., Wang M. (2009). Intrinsic subtype-associated changes in the plasma proteome in breast cancer. Proteom. Clin. Appl..

[9] Zhang F., Chen J.Y. (2013). Breast cancer subtyping from plasma proteins. BMC Med. Genet..

[10] Gajbhiye A., Dabhi R., Taunk K., Jagadeeshaprasad M.G., RoyChoudhury S., Mane A., Bayatigeri S., Chaudhury K., Santra M.K., Rapole S. (2017). Multipronged quantitative proteomics reveals serum proteome alterations in breast cancer intrinsic subtypes. J. Proteome.

[11] Oda M., Makita M., Iwaya K., Akiyama F., Kohno N., Tsuchiya B., Iwase T., Matsubara O. (2012). High levels of DJ 1 protein in nipple fluid of patients with breast cancer. Cancer Sci..

[12] Somlo G., Lau S.K., Frankel P., Hsieh H.B., Liu X., Yang L., Krivacic R., Bruce R.H. (2011). Multiple biomarker expression on circulating tumor cells in comparison to tumor tissues from primary and metastatic sites in patients with locally advanced/inflammatory, and stage IV breast cancer, using a novel detection technology. Breast Cancer Res. Treat..

[13] Wang D.L., Xiao C., Fu G., Wang X., Li L. (2017). Identification of potential serum biomarkers for breast cancer using a functional proteomics technology. Biomark Res..

[14] VanMeter A.J., Camerini S., Polci M.L., Tessitore A., Trivedi N., Heiby M., Kamal Y., Hansen J., Zhou W. (2012). Serum low-molecular-weight protein fractionation for biomarker discovery. Methods Mol. Biol..

[15] Lee P.Y., Osman J., Low T.Y., Jamal R. (2019). Plasma/serum proteomics: Depletion strategies for reducing high-abundance proteins for biomarker discovery. Bioanalysis.

[16] Chertov O., Simpson J.T., Biragyn A., Conrads T.P., Veenstra T.D., Fisher R.J. (2005). Enrichment of low-molecular-weight proteins from biofluids for biomarker discovery. Expert Rev. Proteom..

[17] Zhang L., Lu H., Yang P. (2010). Recent developments of nanoparticle-based enrichment methods for mass spectrometric analysis in proteomic. Sci. China Chem..

[18] Docter D., Westmeier D., Markiewicz M., Stolte S., Knauer S.K., Stauber R.H. (2015). The nanoparticle biomolecule corona: Lessons learned—challenge accepted?. Chem. Soc. Rev..

[19] Lundqvist M., Stigler J., Elia G., Lynch I., Cedervall T., Dawson K.A. (2008). Nanoparticle size and surface properties determine the protein corona with possible implications for biological impacts. Proc. Natl. Acad. Sci. USA.

[20] Mahmoudi M., Lynch I., Ejtehadi M.R., Monopoli M.P., Bombelli F.B., Laurent S. (2011). Protein-nanoparticle interactions: Opportunities and challenges. Chem. Rev..

[21] Monopoli M.P., Walczyk D., Campbell A., Elia G., Lynch I., Baldelli Bombelli F., Dawson K.A. (2011). Physical-chemical aspects of protein corona: Relevance to in vitroand in vivo biological impacts of nanoparticles. J. Am. Chem. Soc..

[22] Laurent S., Burtea C., Thirifays C., Rezaee F., Mahmoudi M. (2013). Significance of cell “observer” and protein source in nanobiosciences. J. Colloid Interface Sci..

[23] Pozzi D., Caracciolo G., Digiacomo L., Colapicchioni V., Palchetti S., Capriotti A.L., Cavaliere C., Zenezini Chiozzi R., Puglisi A., Laganà A. (2015). The biomolecular corona of nanoparticles in circulating biological media. Nanoscale.

[24] Mahmoudi M., Abdelmonem A.M., Behzadi S., Clement J.H., Dutz S., Ejtehadi M.R., Hartmann R., Kantner K., Linne U., Maffre P. (2013). Temperature: The “ignored” factor at the NanoBio interface. ACS Nano..

[25] Tenzer S., Docter D., Kuharev J., Musyanovych A., Fetz V., Hecht R., Schlenk F., Fischer D., Kiouptsi K., Reinhardt C. (2013). Rapid formation of plasma protein corona critically affects nanoparticle pathophysiology. Nat. Nanotechnol..

[26] Núñez C., Chantada-Vázquez M.P., Vázquez-Estévez S. (2018). Inorganic nanoparticles in diagnosis and treatment of breast cancer. J. Biol. Inorg. Chem..

[27] Núñez C., Chantada-Vázquez M.P., Bravo S.B., Vázquez-Estévez S. (2018). Novel functionalized nanomaterials for the effective enrichment of proteins and peptides with post-translational modifications. J. Proteom..

[28] Chantada-Vázquez M.P., Castro A., Bravo S.B., Vázquez-Estévez S., Acea-Nebril B., Núñez C. (2019). Proteomic analysis of the bio-corona formed on the surface of (Au, Ag, Pt)-nanoparticles in human serum. Colloids Surf. B Biointerfaces.

[29] Núñez C. (2019). Blood-based protein biomarkers in breast cancer. Clin. Chim. Acta.

[30] García Vence M., Chantada-Vázquez M.P., Vázquez-Estévez S., Cameselle-Teijeiro J.M., Bravo S.B., Núñez C. (2020). Potential clinical applications of the personalized, disease-specific protein corona on nanoparticles. Clin. Chim. Acta.

[31] Chantada-Vázquez M.P., Castro A., García Vence M., Vázquez-Estévez S., Acea-Nebril B., Calatayud D.G., Jardiel T., Bravo S.B., Núñez C. (2020). Proteomic investigation on bio-corona of Au, Ag and Fe nanoparticles for the discovery of triple negative breast cancer serum protein biomarkers. J. Proteom..

[32] Couto C., Vitorino R., Daniel-da-Silva A.L. (2017). Gold nanoparticles and bioconjugation: A pathway for proteomic applications. Crit. Rev. Biotechnol..

[33] Lopez-Cortés R., Oliveira E., Núñez C., Lodeiro C., Páez de la Cadena M., Fdez-Riverola F., López-Fernández H., Reboiro-Jato M., Glez-Peña D., Capelo J.L. (2012). Fast human serum profiling through chemical depletion coupled to gold-nanoparticle-assisted protein separation. Talanta.

[34] Warder S.E., Tucker L.A., Strelitzer T.J., McKeegan E.M., Meuth J.L., Jung P.M., Saraf A., Singh B., Lai-Zhang J., Gagne G. (2009). Reducing agent-mediated precipitation of high-abundance plasma proteins. Anal. Biochem..

[35] Fernández C., Santos H.M., Ruíz-Romero C., Blanco F.J., Capelo-Martínez J.L. (2011). A comparison of depletion versus equalization for reducing high-abundance proteins in human serum. Electrophoresis.

[36] Candiano G., Bruschi M., Musante L., Santucci L., Ghiggeri G.M., Carnemolla B., Orecchia P., Zardi L., Righetti P.G. (2004). Blue silver: A very sensitive colloidal Coomassie G-250 staining for proteome analysis. Electrophoresis.

[37] Oliveira E., Araújo J.E., Gómez-Meire S., Lodeiro C., Pérez-Melón C., Iglesias-Lamas E., Otero-Glez A., Capelo J.L., Santos H.M. (2014). Proteomics analysis of the peritoneal dialysate effluent reveals the presence of calcium-regulation proteins and acute inflammatory response. Clin. Proteom..

[38] Shilov I.V., Seymour S.L., Patel A.A., Loboda A., Tang W.H., Keating S.P., Hunter C.L., Nuwaysir L.M., Schaeffer D.A. (2007). The paragon algorithm, a next generation search engine that uses sequence temperature values and feature probabilities to identify peptides from tandem mass spectra. Mol. Cell Proteom..

[39] Tang W.H., Shilov I.V., Seymour S.L. (2008). Nonlinear fitting method for determining local false discovery rates from decoy database searches. J. Proteome Res..

[40] Szklarczyk D., Franceschini A., Wyder S., Forslund K., Heller D., Huerta-Cepas J., Simonovic M., Roth A., Santos A., Tsafou K.P. (2015). STRING v10: Protein-protein interaction networks, integrated over the tree of life. Nucleic Acids Res..

[41] Dobrovolskaia M.A., Patri A.K., Zheng J., Clogston J.D., Ayub N., Aggarwal P., Neun B.W., Hall J.B., McNeil S.E. (2009). Interaction of colloidal gold nanoparticles with human blood: Effects on particle size and analysis of plasma protein binding profiles. Nanomedicine.

[42] García-Álvarez R., Hadjidemetriou M., Sánchez-Iglesias A., Liz-Marzán L.M., Kostarelos K. (2018). In vivo formation of protein corona on gold nanoparticles. The effect of size and shape. Nanoscale.

[43] Fry S.A., Sinclair J., Timms J.F., Leathem A.J., Dwek M.V. (2013). A targeted glycoproteomic approach identifies cadherin-5 as a novel biomarker of metastatic breast cancer. Cancer Lett..

[44] Fry S.A., Robertson C.E., Swann R., Dwek M.V. (2016). Cadherin-5: A biomarker for metastatic breast cancer with optimum efficacy in oestrogen receptor-positive breast cancers with vascular invasion. Br. J. Cancer.

[45] Cassatella M.A., Östberg N.K., Tamassia N., Soehnlein O. (2019). Biological Roles of Neutrophil-Derived Granule Proteins and Cytokines. Trends Immunol..

[46] Mollinedo F. (2019). Neutrophil Degranulation, Plasticity, and Cancer Metastasis. Trends Immunol..

[47] Borregaard N., Sørensen O.E., Theilgaard-Mönch K. (2007). Neutrophil granules: A library of innate immunity proteins. Trends Immunol..

[48] Mollinedo F., Pulido R., Lacal P.M., Sánchez-Madrid F. (1991). Mobilization of gelatinase-rich granules as a regulatory mechanism of early functional responses in human neutrophils. Scand. J. Immunol..

[49] Deryugina E.I., Zajac E., Juncker-Jensen A., Kupriyanova T.A., Welter L., Quigley J.P. (2014). Tissue-infiltrating neutrophils constitute the major in vivo source of angiogenesis-inducing MMP-9 in the tumor microenvironment. Neoplasia.

[50] Bundred N.J., Dover M.S., Aluwihare N., Faragher E.B., Morrison J.M. (1993). Smoking and periductal mastitis. BMJ..

[51] Josephy P.D. (1996). The role of peroxidase-catalyzed activation of aromatic amines in breast cancer. Mutagenesis.

[52] Samoszuk M.K., Nguyen V., Gluzman I., Pham J.H. (1996). Occult deposition of eosinophil peroxidase in a subset of human breast carcinomas. Am. J. Pathol..

[53] Panagopoulos V., Leach D.A., Zinonos I., Ponomarev V., Licari G., Liapis V., Ingman W.V., Anderson P., DeNichilo M.O., Evdokiou A. (2017). Inflammatory peroxidases promote breast cancer progression in mice via regulation of the tumour microenvironment. Int. J. Oncol..

[54] Wirthmueller U., Dewald B., Thelen M., Schäfer M.K., Stover C., Whaley K., North J., Eggleton P., Reid K.B., Schwaeble W.J. (1997). Properdin, a positive regulator of complement activation, is released from secondary granules of stimulated peripheral blood neutrophils. J. Immunol..

[55] Wrighl D.G., Gallin J.L., Fauci A.S. (1982). The neutrophil as a secretory organ of host defense. Advances in Host Defense Mechanisms.

[56] Nitto T., Onodera K. (2013). Linkage between coenzyme A metabolism and inflammation: Roles of pantetheinase. J. Pharmacol. Sci..

[57] Bjerrum O.W., Bjerrum O.J., Borregaard N. (1987). Beta 2-microglobulin in neutrophils: An intragranular protein. J. Immunol..

[58] Gebhardt C., Németh J., Angel P. (2006). Hess, S100A8 and S100A9 in inflammation and cancer. J. Biochem. Pharmacol..

[59] Klein T., Levin I., Niska A., Koren R., Gal R., Schachter J., Kfir B., Narinski R., Warchaizer S., Klein B. (1996). Correlation between tumour and serum beta 2m expression in patients with breast cancer. Eur. J. Immunogenet..

[60] Madjd Z., Spendlove I., Pinder S.E., Ellis I.O., Durrant L.G. (2005). Total loss of MHC class I is an independent indicator of good prognosis in breast cancer. Int. J. Cancer.

[61] Scheffer G.L., de Jong M.C., Monks A., Flens M.J., Hose C.D., Izquierdo M.A., Shoemaker R.H., Scheper R.J. (2002). Increased expression of beta 2-microglobulin in multidrug-resistant tumour cells. Br. J. Cancer..

[62] Moon A., Yong H.Y., Song J.I., Cukovic D., Salagrama S., Kaplan D., Putt D., Kim H., Dombkowski A., Kim H.R. (2008). Global gene expression profiling unveils S100A8/A9 as candidate markers in H-ras-mediated human breast epithelial cell invasion. Mol. Cancer Res..

[63] Yin C., Li H., Zhang B., Liu Y., Lu G., Lu S., Sun L., Qi Y., Li X., Chen W. (2013). RAGE-binding S100A8/A9 promotes the migration and invasion of human breast cancer cells through actin polymerization and epithelial-mesenchymal transition. Breast Cancer Res. Treat..

[64] Carlsson H., Petersson S., Enerback C. (2005). Cluster analysis of S100 gene expression and genes correlating to psoriasin (S100A7) expression at different stages of breast cancer development. Int. J. Oncol..

[65] Rhee D.K., Park S.H., Jang Y.K. (2008). Molecular signatures associated with transformation and progression to breast cancer in the isogenic MCF10 model. Genomics.

[66] Bao Y., Wang A., Mo J. (2016). S100A8/A9 is associated with estrogen receptor loss in breast cancer. Oncol. Lett..

[67] Naleskina L.A., Lukianova N.Y., Sobchenko S.O., Storchai D.M., Chekhun V.F. (2016). Lactoferrin expression in breast cancer in relation to biologic properties of tumors and clinical features of disease. Exp. Oncol..

[68] Hoedt E., Chaoui K., Huvent I., Mariller C., Monsarrat B., Burlet-Schiltz O., Pierce A. (2014). SILAC-based proteomic profiling of the human MDA-MB-231 metastatic breast cancer cell line in response to the two antitumoral lactoferrin isoforms: The secreted lactoferrin and the intracellular delta-lactoferrin. PLoS ONE.

[69] Mahanta S., Paul S., Srivastava A., Pastor A., Kundu B., Chaudhuri T.K. (2015). Stable self-assembled nanostructured hen egg white lysozyme exhibits strong anti-proliferative activity against breast cancer cells. Colloids Surf. B Biointerfaces.

[70] Udby L., Cowland J.B., Johnsen A.H., Sorensen O.E., Borregaard N., Kjeldsen L. (2002). An ELISA for SGP28/ CRISP-3, a cysteine-rich secretory protein in human neutrophils, plasma, and exocrine secretions. J. Immunol. Methods.

[71] Wang Y., Sheng N., Xie Y., Chen S., Lu J., Zhang Z., Shan Q., Wu D., Zheng G., Li M. (2019). Low expression of CRISP3 predicts a favorable prognosis in patients with mammary carcinoma. J. Cell Physiol..

[72] Harris J.P., Caleb M.H., South M.A. (1975). Secretory component in human mammary carcinoma. Cancer Res..

[73] Nyberg P., Ylipalosaari M., Sorsa T., Salo T. (2006). Trypsins and their role in carcinoma growth. Exp. Cell Res..

[74] Kehlen A., Lendeckel U., Dralle H., Langner J., Hoang-Vu C. (2003). Biological significance of aminopeptidase N/CD13 in thyroid carcinomas. Cancer Res..

[75] Qian L., Gao X., Huang H., Lu S., Cai Y., Hua Y., Liu Y., Zhang J. (2017). PRSS3 is a prognostic marker in invasive ductal carcinoma of the breast. Oncotarget.

[76] Dixon J., Kaklamanis L., Turley H., Hickson I.D., Leek R.D., Harris A.L., Gatter K.C. (1994). Expression of aminopeptidase-N (CD 13) in normal tissues and malignant neoplasms of epithelial and lymphoid origin. J. Clin. Pathol..

[77] Ranogajec I., Jakić-Razumović J., Puzović V., Gabrilovac J. (2012). Prognostic value of matrix metalloproteinase-2 (MMP-2), matrix metalloproteinase-9 (MMP-9) and aminopeptidase N/CD13 in breast cancer patients. Med. Oncol..

[78] Bai Z., Ye Y., Liang B., Feng X., Zhang H., Zhang Y., Peng J., Shen D., Cui Z., Zhang Z. (2011). Proteomics-based identification of a group of apoptosis-related proteins and biomarkers in gastric cancer. Int. J. Oncol..

[79] Lee D.H., Chung K., Song J.A., Kim T.H., Kang H., Huh J.H., Jung S.G., Ko J.J., An H.J. (2010). Proteomic identification of paclitaxel-resistance associated hnRNP A2 and GDI 2 proteins in human ovarian cancer cells. J. Proteome Res..

[80] Zhang P., Garnett J., Creighton C.J., Al Sannaa G.A., Igram D.R., Lazar A., Liu X., Liu C., Pollock R.E. (2014). EZH2-miR-30d-KPNB1 pathway regulates malignant peripheral nerve sheath tumour cell survival and tumourigenesis. J. Pathol..

[81] Hendrix A., Braems G., Bracke M., Seabra M., Gahl W., De Wever O., Westbroek W. (2010). The secretory small GTPase Rab27B as a marker for breast cancer progression. Oncotarget.

[82] Yang P.S., Yin P.H., Tseng L.M., Yang C.H., Hsu C.Y., Lee M.Y., Horng C.F., Chi C.W. (2011). Rab5A is associated with axillary lymph node metastasis in breast cancer patients. Cancer Sci..

[83] van der Watt P.J., Stowell C.L., Leaner V.D. (2013). The nuclear import receptor Kpnβ1 and its potential as an anticancer therapeutic target. Crit. Rev. Eukaryot. Gene Expr..

[84] de la Cruz-Merino L., Barco-Sánchez A., Henao Carrasco F., Nogales Fernández E., Vallejo Benítez A., Brugal Molina J., Martínez Peinado A., Grueso López A., Ruiz Borrego M., Codes Manuel de Villena M. (2013). New insights into the role of the immune microenvironment in breast carcinoma. Clin. Dev. Immunol..

[85] Oda K., Matsuoka Y., Funahashi A., Kitano H. (2005). A comprehensive pathway map of epidermal growth factor receptor signaling. Mol. Syst. Biol..

[86] Sobhani N., Fan C., Flores-Villanueva P.O., Generali D., Li Y. (2020). The fibroblast growth factor receptors in breast cancer: From oncogenesis to better treatments. Int. J. Mol. Sci..

