# Supplementary material for: Identification of a Profile of Neutrophil-Derived Granule Proteins in the Surface of Gold Nanoparticles after Their Interaction with Human Breast Cancer Sera
Source: Nanomaterials (Basel). 2020 Jun 23;10(6):1223. doi: 10.3390/nano10061223 (PMC7353125; doi:10.3390/nano10061223)
Supplement: Supplementary file 1 [file nanomaterials-10-01223-s001.pdf]

Figure S1. TEM image of AuNPs@citrate in aqueous phase and the characterization data.

**AuNPs@citrate (nm)**			
1	7.94	21	10.35
2	8.37	22	10.38
3	8.38	23	10.41
4	8.60	24	10.44
5	8.78	25	10.56
6	8.78	26	10.65
7	8.87	27	10.65
8	9.17	28	10.68
9	9.19	29	10.80
10	9.41	30	10.81
11	9.49	31	10.83
12	9.49	32	10.85
13	9.55	33	10.86
14	9.62	34	10.86
15	9.82	35	10.88
16	9.84	36	10.95
17	9.89	37	10.96
18	9.89	38	10.99
19	9.99	39	11.48
20	10.03	40	11.50
Count	40		
Mean	10.02		
Minimum	7.94		
Maximum	11.50		
Standard Deviation	0.91		

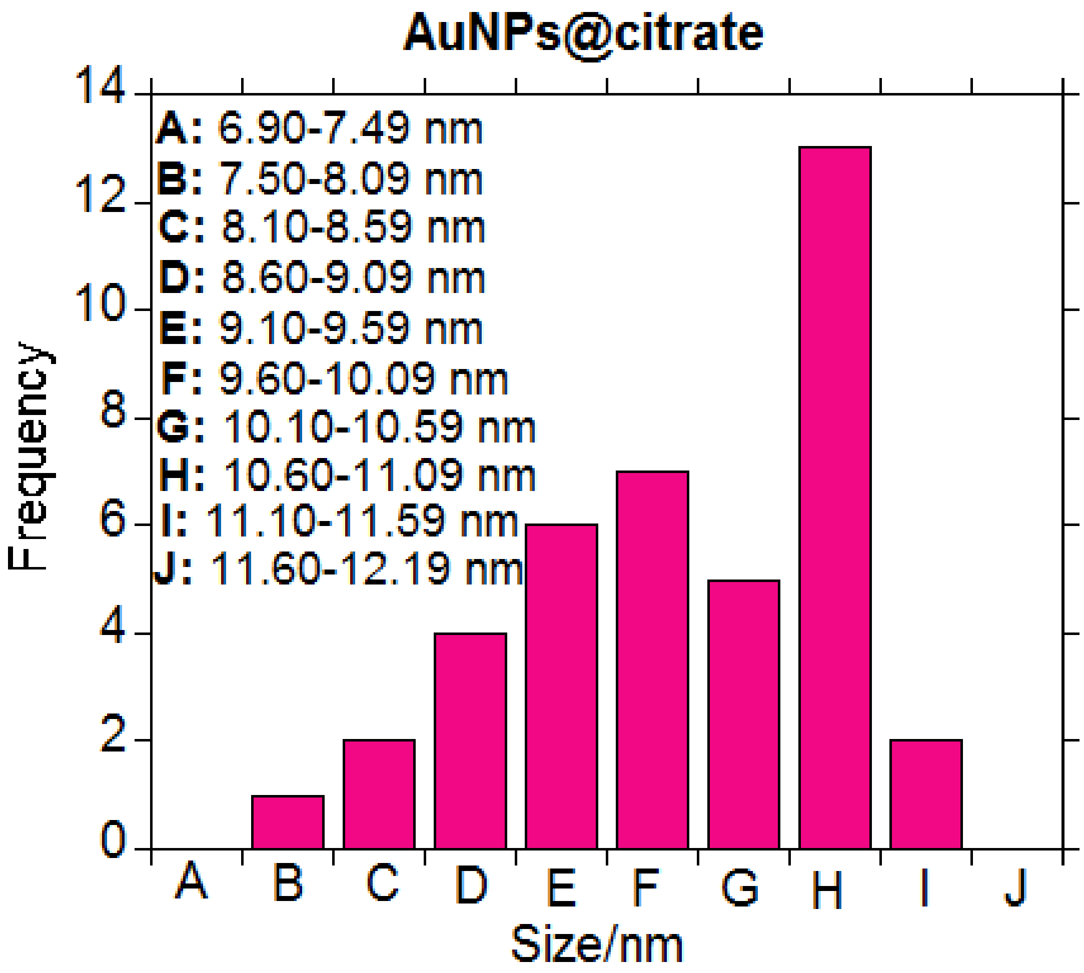

AuNPs@citrate
A: 6.90-7.49 nm
B: 7.50-8.09 nm
C: 8.10-8.59 nm
D: 8.60-9.09 nm
E: 9.10-9.59 nm
F: 9.60-10.09 nm
G: 10.10-10.59 nm
H: 10.60-11.09 nm
I: 11.10-11.59 nm
J: 11.60-12.19 nm
Frequency
0
2
4
6
8
10
12
14
A
B
C
D
E
F
G
H
I
J
Size/nm

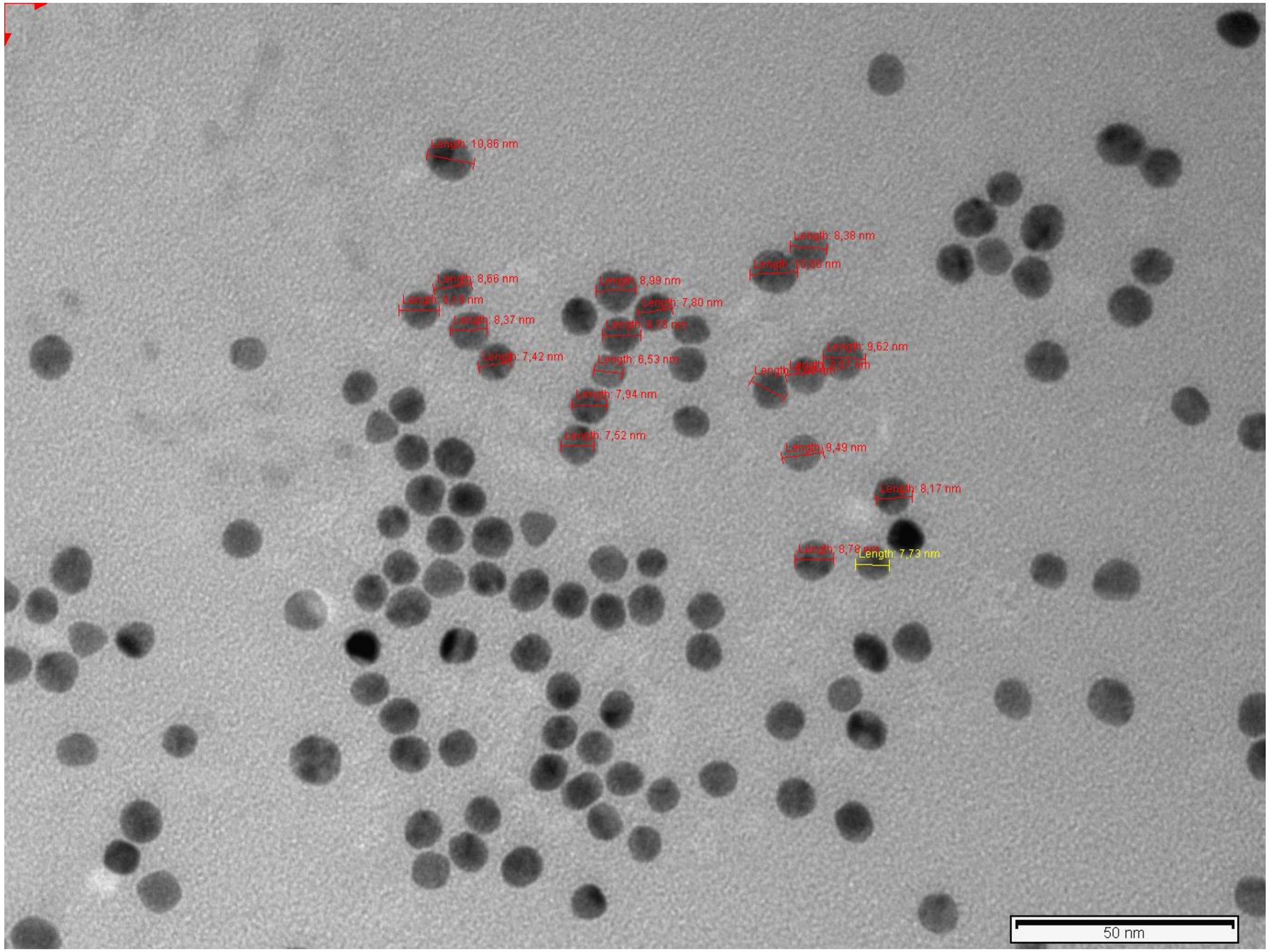
Length: 10,86 nm
Length: 8,38 nm
Length: 8,66 nm
Length: 8,99 nm
Length: 7,80 nm
Length: 8,37 nm
Length: 7,42 nm
Length: 6,53 nm
Length: 9,62 nm
Length: 7,94 nm
Length: 7,52 nm
Length: 9,49 nm
Length: 8,17 nm
Length: 7,73 nm
50 nm

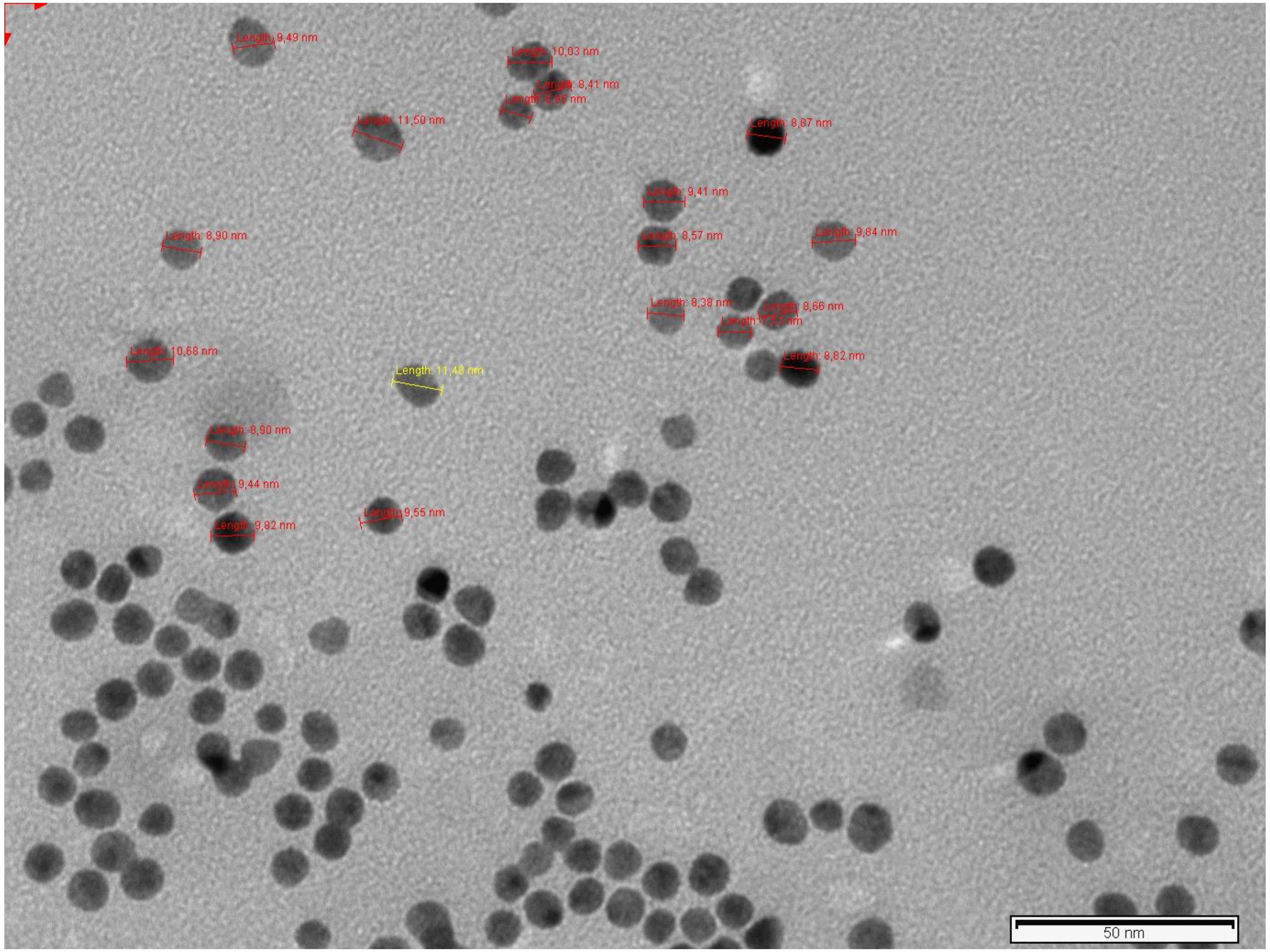
Length: 9,49 nm
Length: 10,03 nm
Length: 8,41 nm
Length: 11,50 nm
Length: 8,87 nm
Length: 9,41 nm
Length: 8,90 nm
Length: 8,57 nm
Length: 9,84 nm
Length: 8,38 nm
Length: 8,66 nm
Length: 10,68 nm
Length: 8,82 nm
Length: 11,48 nm
Length: 8,90 nm
Length: 9,44 nm
Length: 9,55 nm
Length: 9,82 nm
50 nm

Figure S2. TEM image of AuNPs@PC-controls in aqueous phase and the characterization data.

AuNPs@PC-controls (nm)			
1	10.52	16	12.29
2	10.73	17	12.33
3	10.85	18	12.39
4	10.97	19	12.46
5	10.99	20	12.63
6	11.03	21	12.65
7	11.32	22	12.72
8	11.50	23	12.77
9	11.60	24	12.90
10	11.72	25	12.94
11	11.89	26	13.04
12	11.90	27	13.06
13	11.94	28	13.35
14	12.03	29	13.54
15	12.03	30	15.05
Count	30		
Mean	12.17		
Minimum	10.52		
Maximum	15.05		
Standard Deviation	0.98		

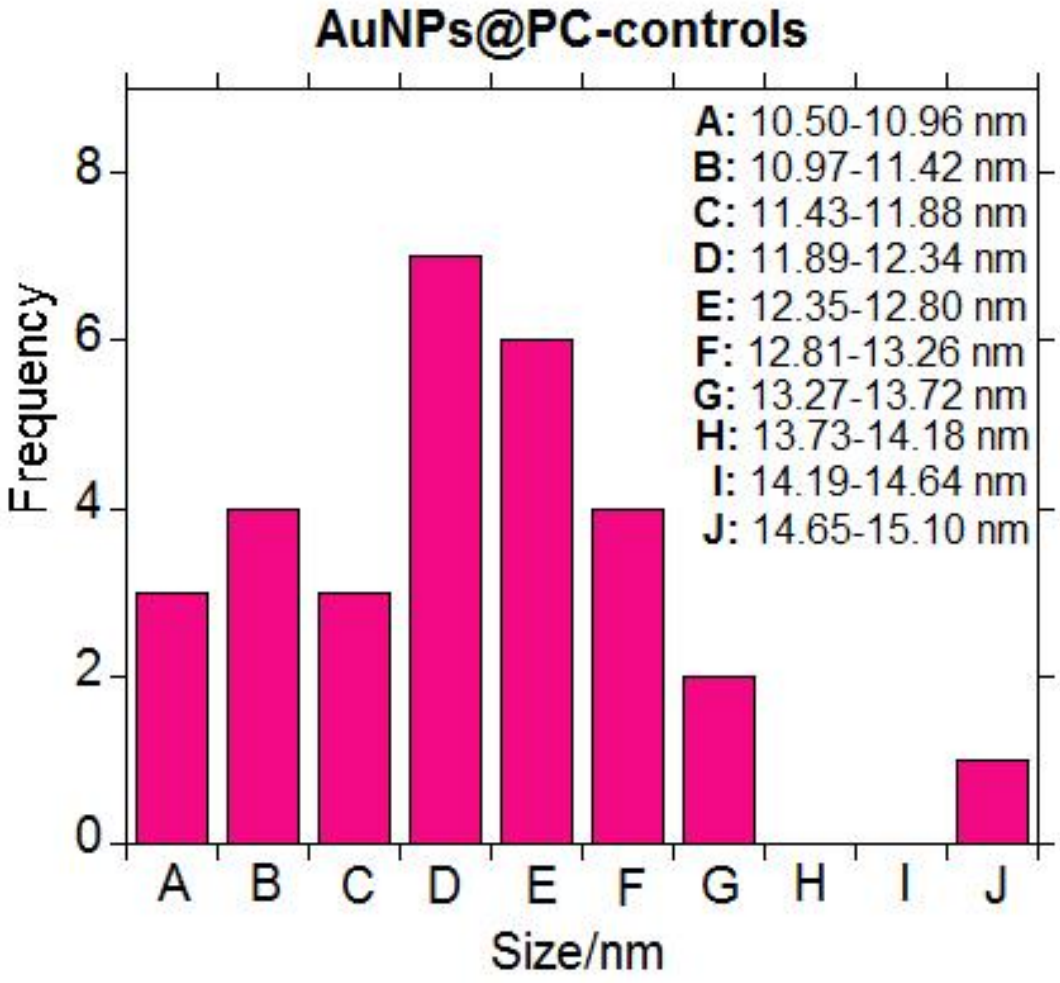
AuNPs@PC-controls
A: 10.50-10.96 nm
B: 10.97-11.42 nm
C: 11.43-11.88 nm
D: 11.89-12.34 nm
E: 12.35-12.80 nm
F: 12.81-13.26 nm
G: 13.27-13.72 nm
H: 13.73-14.18 nm
I: 14.19-14.64 nm
J: 14.65-15.10 nm
Frequency
0
2
4
6
8
A B C D E F G H I J
Size/nm

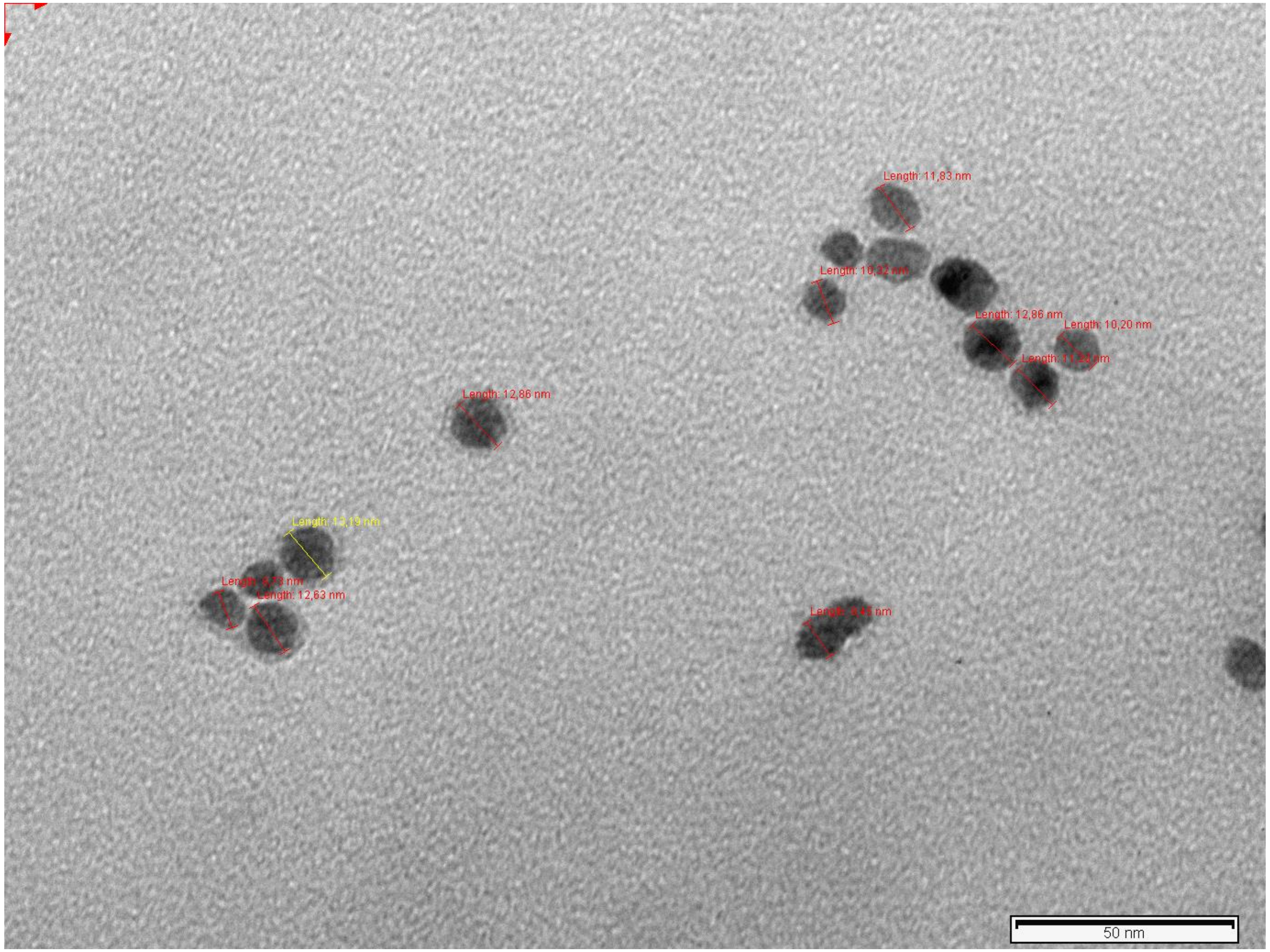
Length: 11,83 nm
Length: 10,32 nm
Length: 12,86 nm
Length: 10,20 nm
Length: 12,86 nm
Length: 12,63 nm
50 nm

Figure S3. TEM image of AuNPs@PC-BC in aqueous phase and the characterization data.

**AuNPs@PC-BC (nm)**			
1	10.12	16	12.32
2	10.71	17	12.32
3	11.09	18	12.38
4	11.11	19	12.47
5	11.31	20	12.51
6	11.45	21	12.51
7	11.45	22	12.53
8	11.50	23	12.61
9	11.74	24	12.61
10	11.79	25	12.62
11	11.96	26	12.95
12	11.98	27	13.04
13	11.98	28	13.31
14	12.20	29	13.51
15	12.32	30	13.73
Count	30		
Mean	12.14		
Minimum	10.12		
Maximum	13.73		
Standard Deviation	0.82		

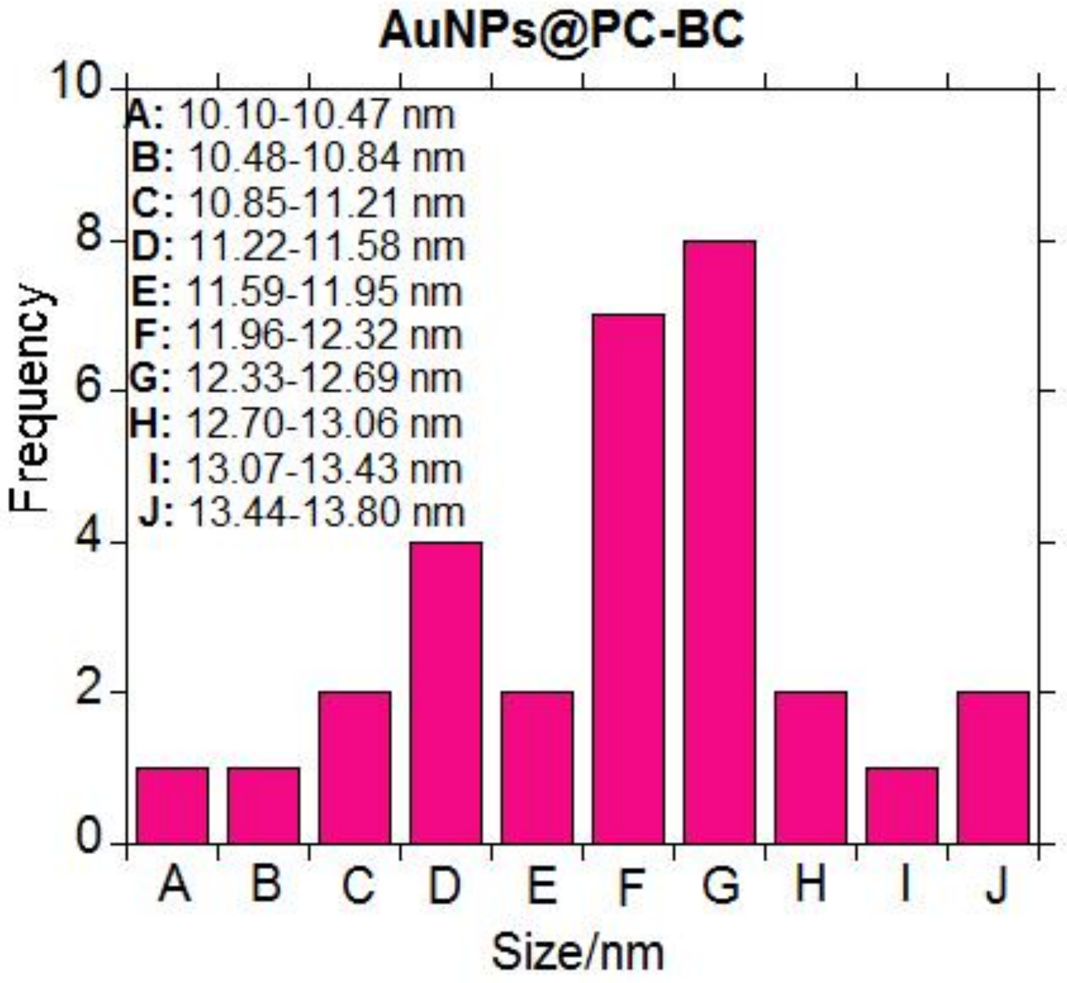
AuNPs@PC-BC
A: 10.10-10.47 nm
B: 10.48-10.84 nm
C: 10.85-11.21 nm
D: 11.22-11.58 nm
E: 11.59-11.95 nm
F: 11.96-12.32 nm
G: 12.33-12.69 nm
H: 12.70-13.06 nm
I: 13.07-13.43 nm
J: 13.44-13.80 nm
Frequency
Size/nm
0
2
4
6
8
10
A
B
C
D
E
F
G
H
I
J

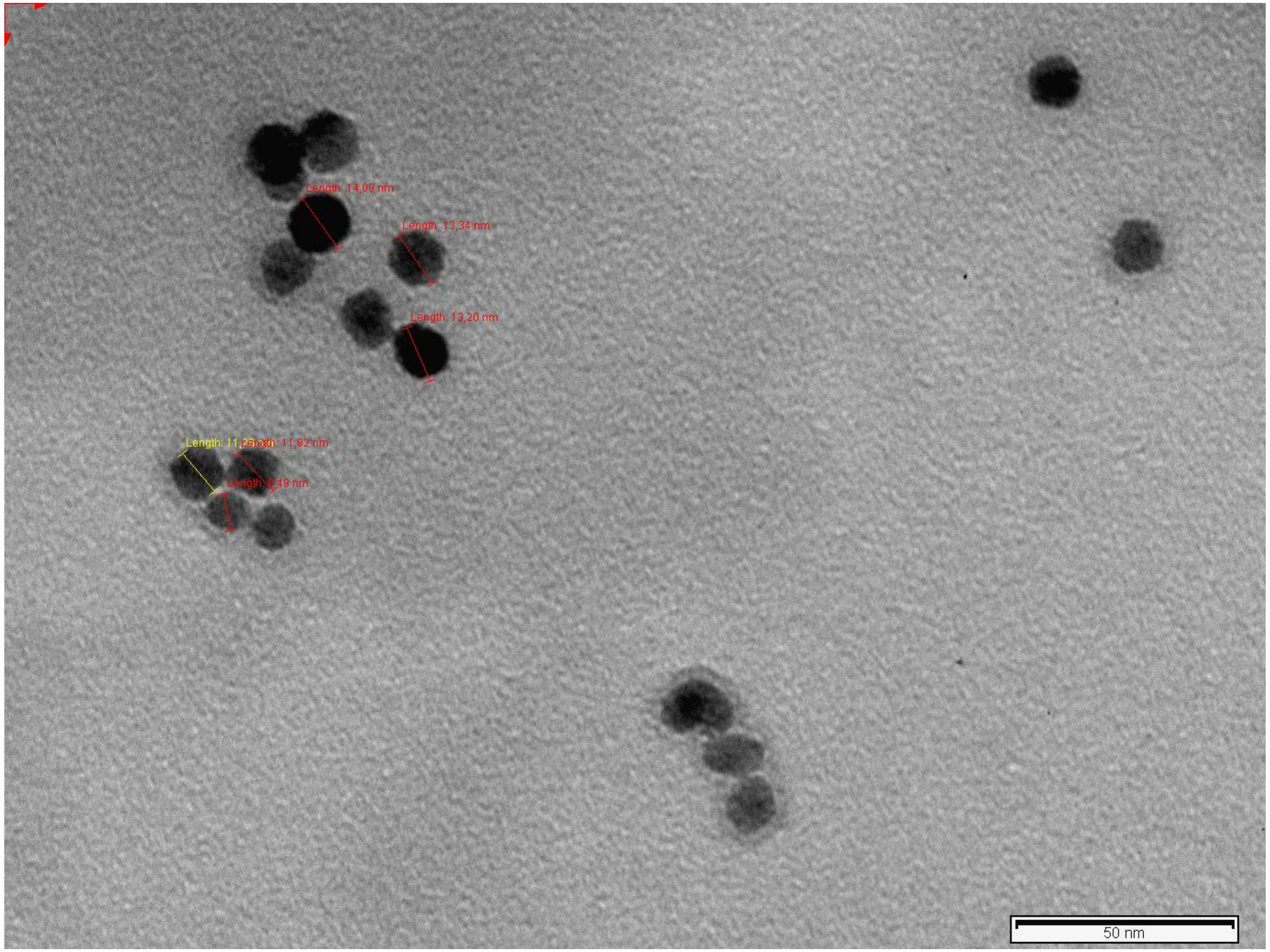
Length: 14,09 nm
Length: 13,34 nm
Length: 13,20 nm
Length: 11,82 nm
Length: 8,49 nm
50 nm

Figure S4. Protein-protein interaction network map of the 194 genes encoded differentially proteins identified in the AuNPs-protein corona after the interaction of AuNPs (10.02 ± 0.91 nm) (30 min, incubation) with serum samples belonging to 42 breast cancer patients.

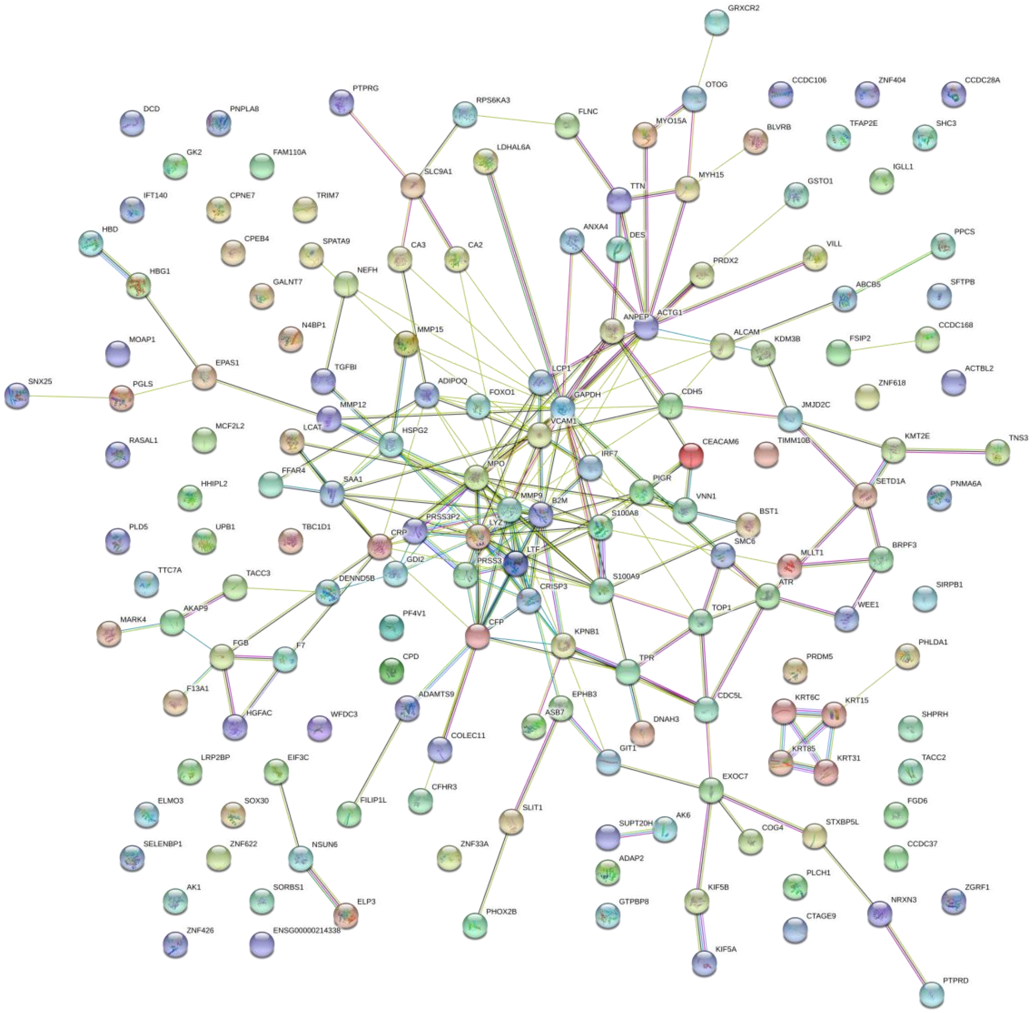

Table S1. Number of proteins identified in the protein corona formed after the interaction of AuNPs (10.02 ± 0.91 nm) (30 min, incubation) with serum samples belonging to 42 healthy women (HC) and 42 breast cancer patients (BC) with different subtypes: luminal A (n = 11), luminal B HER2 negative (n = 10), luminal B HER2 positive (n = 7), HER2 positive (n = 6), and triple negative (n = 8).

**Fraction** / **Classification**	**AuNPs-protein corona**		
	**Number of proteins identified**		
	**Total**	**Common**	**Uniques**
Control (n = 42)	350	252	98
Luminal A (n = 11)	324		72
Control (n = 42)	350	247	103
Luminal B HER2 negative (n = 10)	329		82
Control (n = 42)	359	235	115
Luminal B HER2 positive (n = 7)	294		59
Control (n = 42)	350	207	207
HER2 positive (n = 6)	243		36
Control (n = 42)	350	237	113
Triple negative (n = 8)	296		59

Table S2. Proteins identified in the protein corona formed after the interaction of AuNPs (10.02 ± 0.91 nm) (30 min, incubation) with serum samples belonging to 42 healthy women (HC) and 42 breast cancer patients (BC).

Protein Name	UniProt Name	HC	BC
Immunoglobulin lambda variable 4-69	LV469_HUMAN	X	X
Immunoglobulin lambda variable 8-61	LV861_HUMAN	X	X
Immunoglobulin lambda variable 4-60	LV460_HUMAN	X	X
Immunoglobulin lambda variable 3-10	LV310_HUMAN	X	X
Immunoglobulin heavy variable 3-64	HV364_HUMAN	X	X
Immunoglobulin heavy variable 4-4	HV404_HUMAN	X	X
Immunoglobulin kappa variable 1-27	KV127_HUMAN	X	X
Immunoglobulin kappa variable 3D-15	KVD15_HUMAN	X	X
Immunoglobulin kappa variable 1D-8	KVD08_HUMAN	X	X
Immunoglobulin heavy variable 3-49	HV349_HUMAN	X	X
Immunoglobulin heavy variable 6-1	HV601_HUMAN	X	X
Immunoglobulin heavy variable 3-15	HV315_HUMAN	X	X
Immunoglobulin heavy variable 2-26	HV226_HUMAN	X	X
Immunoglobulin heavy variable 3-74	HV374_HUMAN	X	X
Immunoglobulin heavy variable 3-43	HV343_HUMAN	X	X
Immunoglobulin lambda variable 9-49	LV949_HUMAN	X	X
Immunoglobulin heavy variable 3-72	HV372_HUMAN	X	X
Immunoglobulin kappa variable 3D-20	KVD20_HUMAN	X	X
Immunoglobulin heavy variable 1-3	HV103_HUMAN	X	X
Immunoglobulin heavy variable 4-28	HV428_HUMAN	X	X
Immunoglobulin heavy variable 5-51	HV551_HUMAN	X	X
Immunoglobulin heavy variable 3-66	HV366_HUMAN	X	X

Immunoglobulin heavy variable 2-70D	HV70D_HUMAN	X	X
Immunoglobulin kappa variable 2-24	KV224_HUMAN	X	X
Immunoglobulin kappa variable 1-9	KV109_HUMAN	X	X
Immunoglobulin kappa variable 1-6	KV106_HUMAN	X	X
Immunoglobulin lambda variable 5-39	LV539_HUMAN	X	X
Immunoglobulin heavy variable 3-64D	HV64D_HUMAN	X	X
Immunoglobulin heavy variable 5-10-1	HV5X1_HUMAN	X	X
Immunoglobulin lambda constant 7	IGLC7_HUMAN	X	X
Transmembrane protein 223	TM223_HUMAN	X	X
Immunoglobulin kappa variable 2-29	KV229_HUMAN	X	X
Immunoglobulin lambda-like polypeptide 5	IGLL5_HUMAN	X	X
Mannan-binding lectin serine protease 2	MASP2_HUMAN	X	X
Sulfhydryl oxidase 1	QSOX1_HUMAN	X	X
Apolipoprotein L1	APOL1_HUMAN	X	X
CD5 antigen-like	CD5L_HUMAN	X	X
Fukutin	FKTN_HUMAN	X	X
Ficolin-3	FCN3_HUMAN	X	X
Attractin	ATRN_HUMAN	X	X
Apolipoprotein M	APOM_HUMAN	X	X
Keratin, type II cytoskeletal 75	K2C75_HUMAN	X	X
Apoptosis-inducing factor 1, mitochondrial	AIFM1_HUMAN	X	X
L-lactate dehydrogenase A chain	LDHA_HUMAN	X	X
Ceruloplasmin	CERU_HUMAN	X	X
Prothrombin	THRB_HUMAN	X	X
Complement C1r subcomponent	C1R_HUMAN	X	X
Haptoglobin	HPT_HUMAN	X	X
Haptoglobin-related protein	HPTR_HUMAN	X	X

Coagulation factor IX	FA9_HUMAN	X	X
Coagulation factor X	FA10_HUMAN	X	X
Complement factor D	CFAD_HUMAN	X	X
Plasminogen	PLMN_HUMAN	X	X
Coagulation factor XII	FA12_HUMAN	X	X
Complement factor B	CFAB_HUMAN	X	X
Carbonic anhydrase 1	CAH1_HUMAN	X	X
Antithrombin-III	ANT3_HUMAN	X	X
Alpha-1-antitrypsin	A1AT_HUMAN	X	X
Alpha-1-antichymotrypsin	AACT_HUMAN	X	X
Angiotensinogen	ANGT_HUMAN	X	X
Alpha-2-macroglobulin	A2MG_HUMAN	X	X
Complement C3	CO3_HUMAN	X	X
Complement C5	CO5_HUMAN	X	X
Kininogen-1	KNG1_HUMAN	X	X
Immunoglobulin J chain	IGJ_HUMAN	X	X
Immunoglobulin kappa variable 1-33	KV133_HUMAN	X	X
Immunoglobulin kappa variable 1-17	KV117_HUMAN	X	X
Immunoglobulin kappa variable 1-5	KV105_HUMAN	X	X
Immunoglobulin kappa variable 1D-12	KVD12_HUMAN	X	X
Immunoglobulin kappa variable 2D-28	KVD28_HUMAN	X	X
Immunoglobulin kappa variable 3-20	KV320_HUMAN	X	X
Immunoglobulin kappa variable 3-15	KV315_HUMAN	X	X
Immunoglobulin lambda variable 1-47	LV147_HUMAN	X	X
Immunoglobulin lambda variable 1-51	LV151_HUMAN	X	X
Immunoglobulin lambda variable 1-40	LV140_HUMAN	X	X
Immunoglobulin lambda variable 2-23	LV223_HUMAN	X	X

Immunoglobulin lambda variable 2-11	LV211_HUMAN	X	X
Immunoglobulin lambda variable 2-8	LV208_HUMAN	X	X
Immunoglobulin lambda variable 3-19	LV319_HUMAN	X	X
Immunoglobulin lambda variable 3-25	LV325_HUMAN	X	X
Immunoglobulin lambda variable 3-27	LV327_HUMAN	X	X
Immunoglobulin heavy variable 1-69	HV169_HUMAN	X	X
Immunoglobulin heavy variable 1-46	HV146_HUMAN	X	X
Immunoglobulin heavy variable 3-23	HV323_HUMAN	X	X
Immunoglobulin heavy variable 3-7	HV307_HUMAN	X	X
Immunoglobulin heavy variable 3-9	HV309_HUMAN	X	X
Immunoglobulin heavy variable 2-5	HV205_HUMAN	X	X
Immunoglobulin heavy variable 4-59	HV459_HUMAN	X	X
Immunoglobulin kappa constant	IGKC_HUMAN	X	X
Immunoglobulin heavy constant gamma 1	IGHG1_HUMAN	X	X
Immunoglobulin heavy constant gamma 2	IGHG2_HUMAN	X	X
Immunoglobulin heavy constant gamma 3	IGHG3_HUMAN	X	X
Immunoglobulin heavy constant gamma 4	IGHG4_HUMAN	X	X
Immunoglobulin heavy constant mu	IGHM_HUMAN	X	X
Immunoglobulin heavy constant alpha 1	IGHA1_HUMAN	X	X
Immunoglobulin heavy constant alpha 2	IGHA2_HUMAN	X	X
Immunoglobulin heavy constant delta	IGHD_HUMAN	X	X
Keratin, type I cytoskeletal 14	K1C14_HUMAN	X	X
Apolipoprotein A-I	APOA1_HUMAN	X	X
Apolipoprotein E	APOE_HUMAN	X	X
Apolipoprotein A-II	APOA2_HUMAN	X	X
Apolipoprotein C-I	APOC1_HUMAN	X	X
Apolipoprotein C-II	APOC2_HUMAN	X	X

Apolipoprotein C-III	APOC3_HUMAN	X	X
Fibrinogen alpha chain	FIBA_HUMAN	X	X
Fibrinogen gamma chain	FIBG_HUMAN	X	X
Serum amyloid P-component	SAMP_HUMAN	X	X
Complement C1q subcomponent subunit A	C1QA_HUMAN	X	X
Complement C1q subcomponent subunit B	C1QB_HUMAN	X	X
Complement C1q subcomponent subunit C	C1QC_HUMAN	X	X
Complement component C9	CO9_HUMAN	X	X
Beta-2-glycoprotein 1	APOH_HUMAN	X	X
Leucine-rich alpha-2-glycoprotein	A2GL_HUMAN	X	X
Fibronectin	FINC_HUMAN	X	X
Retinol-binding protein 4	RET4_HUMAN	X	X
Protein AMBP	AMBP_HUMAN	X	X
Alpha-1-acid glycoprotein 1	A1AG1_HUMAN	X	X
Alpha-2-HS-glycoprotein	FETUA_HUMAN	X	X
Transthyretin	TTHY_HUMAN	X	X
Serum albumin	ALBU_HUMAN	X	X
Vitamin D-binding protein	VTDB_HUMAN	X	X
Platelet basic protein	CXCL7_HUMAN	X	X
Transferrin receptor protein 1	TFR1_HUMAN	X	X
Serotransferrin	TRFE_HUMAN	X	X
Hemopexin	HEMO_HUMAN	X	X
Plasma kallikrein	KLKB1_HUMAN	X	X
C4b-binding protein alpha chain	C4BPA_HUMAN	X	X
Vitronectin	VTNC_HUMAN	X	X
Vitamin K-dependent protein C	PROC_HUMAN	X	X
Apolipoprotein B-100	APOB_HUMAN	X	X

Histidine-rich glycoprotein	HRG_HUMAN	X	X
Immunoglobulin lambda variable 7-43	LV743_HUMAN	X	X
Alpha-1B-glycoprotein	A1BG_HUMAN	X	X
Keratin, type II cytoskeletal 6B	K2C6B_HUMAN	X	X
Keratin, type II cytoskeletal 1	K2C1_HUMAN	X	X
von Willebrand factor	VWF_HUMAN	X	X
Sex hormone-binding globulin	SHBG_HUMAN	X	X
Immunoglobulin kappa variable 1D-39	KVD39_HUMAN	X	X
Immunoglobulin kappa variable 3-11	KV311_HUMAN	X	X
Apolipoprotein D	APOD_HUMAN	X	X
Plasma serine protease inhibitor	IPSP_HUMAN	X	X
Plasma protease C1 inhibitor	IC1_HUMAN	X	X
Complement factor I	CFAI_HUMAN	X	X
Coagulation factor XIII B chain	F13B_HUMAN	X	X
Tetranectin	TETN_HUMAN	X	X
Thyroxine-binding globulin	THBG_HUMAN	X	X
Heparin cofactor 2	HEP2_HUMAN	X	X
Cholinesterase	CHLE_HUMAN	X	X
Immunoglobulin kappa variable 2-30	KV230_HUMAN	X	X
Immunoglobulin kappa variable 4-1	KV401_HUMAN	X	X
Immunoglobulin heavy variable 4-34	HV434_HUMAN	X	X
Gelsolin	GELS_HUMAN	X	X
Complement C2	CO2_HUMAN	X	X
Apolipoprotein A-IV	APOA4_HUMAN	X	X
L-lactate dehydrogenase B chain	LDHB_HUMAN	X	X
Vitamin K-dependent protein S	PROS_HUMAN	X	X
Complement component C8 alpha chain	CO8A_HUMAN	X	X

Complement component C8 beta chain	CO8B_HUMAN	X	X
Platelet glycoprotein Ib alpha chain	GP1BA_HUMAN	X	X
Complement component C8 gamma chain	CO8G_HUMAN	X	X
Trypsin-1	TRY1_HUMAN	X	X
Thrombospondin-1	TSP1_HUMAN	X	X
Corticosteroid-binding globulin	CBG_HUMAN	X	X
Apolipoprotein(a)	APOA_HUMAN	X	X
Monocyte differentiation antigen CD14	CD14_HUMAN	X	X
Complement factor H	CFAH_HUMAN	X	X
Alpha-2-antiplasmin	A2AP_HUMAN	X	X
Keratin, type I cytoskeletal 16	K1C16_HUMAN	X	X
Dopamine beta-hydroxylase	DOPO_HUMAN	X	X
SPARC	SPRC_HUMAN	X	X
Complement C1s subcomponent	C1S_HUMAN	X	X
Complement C4-A	CO4A_HUMAN	X	X
Complement C4-B	CO4B_HUMAN	X	X
Serum amyloid A-2 protein	SAA2_HUMAN	X	X
Immunoglobulin lambda constant 2	IGLC2_HUMAN	X	X
Immunoglobulin lambda constant 3	IGLC3_HUMAN	X	X
Immunoglobulin heavy variable 1-8	HV108_HUMAN	X	X
Immunoglobulin heavy variable 3-30-3	HVC33_HUMAN	X	X
Immunoglobulin heavy variable 3-30-5	HV335_HUMAN	X	X
Immunoglobulin heavy variable 3-43D	HV43D_HUMAN	X	X
Immunoglobulin heavy variable 4-38-2	HVD82_HUMAN	X	X
Immunoglobulin kappa variable 1-13	KV113_HUMAN	X	X
Complement component C7	CO7_HUMAN	X	X
Clusterin	CLUS_HUMAN	X	X

Solute carrier family 2, facilitated glucose transporter member 2	GTR2_HUMAN	X	X
Cholesteryl ester transfer protein	CETP_HUMAN	X	X
Coagulation factor V	FA5_HUMAN	X	X
Lysosome-associated membrane glycoprotein 2	LAMP2_HUMAN	X	X
Keratin, type I cytoskeletal 10	K1C10_HUMAN	X	X
Keratin, type II cytoskeletal 5	K2C5_HUMAN	X	X
Complement component C6	CO6_HUMAN	X	X
L-selectin	LYAM1_HUMAN	X	X
Carboxypeptidase N catalytic chain	CBPN_HUMAN	X	X
CD44 antigen	CD44_HUMAN	X	X
Lipopolysaccharide-binding protein	LBP_HUMAN	X	X
Alpha-1-acid glycoprotein 2	A1AG2_HUMAN	X	X
Inter-alpha-trypsin inhibitor heavy chain H2	ITIH2_HUMAN	X	X
Inter-alpha-trypsin inhibitor heavy chain H1	ITIH1_HUMAN	X	X
Pregnancy zone protein	PZP_HUMAN	X	X
C4b-binding protein beta chain	C4BPB_HUMAN	X	X
Glutathione peroxidase 3	GPX3_HUMAN	X	X
Carboxypeptidase N subunit 2	CPN2_HUMAN	X	X
Vitamin K-dependent protein Z	PROZ_HUMAN	X	X
Fibulin-1	FBLN1_HUMAN	X	X
Zinc-alpha-2-glycoprotein	ZA2G_HUMAN	X	X
Hepatocyte growth factor-like protein	HGFL_HUMAN	X	X
Serum paraoxonase/arylesterase 1	PON1_HUMAN	X	X
Kallistatin	KAIN_HUMAN	X	X
Mannosyl-oligosaccharide 1,2-alpha-mannosidase IA	MA1A1_HUMAN	X	X

Thrombospondin-4	TSP4_HUMAN	X	X
Keratin, type I cytoskeletal 9	K1C9_HUMAN	X	X
Serum amyloid A-4 protein	SAA4_HUMAN	X	X
Insulin-like growth factor-binding protein complex acid labile subunit	ALS_HUMAN	X	X
Keratin, type II cytoskeletal 2 epidermal	K22E_HUMAN	X	X
Pigment epithelium-derived factor	PEDF_HUMAN	X	X
Complement factor H-related protein 2	FHR2_HUMAN	X	X
Platelet glycoprotein V	GPV_HUMAN	X	X
Biotinidase	BTD_HUMAN	X	X
Afamin	AFAM_HUMAN	X	X
Mannan-binding lectin serine protease 1	MASP1_HUMAN	X	X
Cartilage oligomeric matrix protein	COMP_HUMAN	X	X
Selenoprotein P	SEPP1_HUMAN	X	X
Cathelicidin antimicrobial peptide	CAMP_HUMAN	X	X
Lumican	LUM_HUMAN	X	X
Alpha-N-acetylglucosaminidase	ANAG_HUMAN	X	X
Apolipoprotein C-IV	APOC4_HUMAN	X	X
Phospholipid transfer protein	PLTP_HUMAN	X	X
Hemoglobin subunit beta	HBB_HUMAN	X	X
Hemoglobin subunit alpha	HBA_HUMAN	X	X
Phosphatidylinositol-glycan-specific phospholipase D	PHLD_HUMAN	X	X
Immunoglobulin lambda variable 3-21	LV321_HUMAN	X	X
Complement factor H-related protein 1	FHR1_HUMAN	X	X
Inter-alpha-trypsin inhibitor heavy chain H3	ITIH3_HUMAN	X	X
Galectin-3-binding protein	LG3BP_HUMAN	X	X

EGF-containing fibulin-like extracellular matrix protein 1	FBLN3_HUMAN	X	X
Platelet-activating factor acetylhydrolase	PAFA_HUMAN	X	X
Lymphocyte cytosolic protein 2	LCP2_HUMAN	X	X
Secreted phosphoprotein 24	SPP24_HUMAN	X	X
Apolipoprotein F	APOF_HUMAN	X	X
UDP-glucose 4-epimerase	GALE_HUMAN	X	X
Hyaluronan-binding protein 2	HABP2_HUMAN	X	X
Keratin, type II cuticular Hb1	KRT81_HUMAN	X	X
Inter-alpha-trypsin inhibitor heavy chain H4	ITIH4_HUMAN	X	X
Serum paraoxonase/lactonase 3	PON3_HUMAN	X	X
Ficolin-2	FCN2_HUMAN	X	X
Extracellular matrix protein 1	ECM1_HUMAN	X	X
Brefeldin A-inhibited guanine nucleotide-exchange protein 3	BIG3_HUMAN	X	X
Transmembrane protein 198	TM198_HUMAN	X	X
HAUS augmin-like complex subunit 3	HAUS3_HUMAN	X	X
Peptidase inhibitor 16	PI16_HUMAN	X	X
ATP-binding cassette sub-family F member 1	ABCF1_HUMAN	X	X
Dynein assembly factor 1, axonemal	DAAF1_HUMAN	X	X
Protein Shroom3	SHRM3_HUMAN	X	X
Complement factor H-related protein 4	FHR4_HUMAN	X	X
Proteoglycan 4	PRG4_HUMAN	X	X
Carboxypeptidase B2	CBPB2_HUMAN	X	X
RBPJ-interacting and tubulin-associated protein 1	RITA1_HUMAN	X	X
Beta-Ala-His dipeptidase	CNDP1_HUMAN	X	X
N-acetylmuramoyl-L-alanine amidase	PGRP2_HUMAN	X	X

Lysosomal-trafficking regulator	LYST_HUMAN	X	X
Endophilin-A3	SH3G3_HUMAN	X	X
Tudor domain-containing protein 1	TDRD1_HUMAN	X	X
E3 ubiquitin-protein ligase TRIM4	TRIM4_HUMAN	X	X
Zinc finger protein 350	ZN350_HUMAN	X	X
Adipocyte plasma membrane-associated protein	APMAP_HUMAN	X	X
Interleukin-1 receptor accessory protein	IL1AP_HUMAN	X	X
Cartilage acidic protein 1	CRAC1_HUMAN	X	X
Complement C1r subcomponent-like protein	C1RL_HUMAN	X	X
Fetuin-B	FETUB_HUMAN	X	X
Prenylcysteine oxidase 1	PCYOX_HUMAN	X	X
Protein Z-dependent protease inhibitor	ZPI_HUMAN	X	X
Mediator of RNA polymerase II transcription subunit 23	MED23_HUMAN	X	X
Multiple inositol polyphosphate phosphatase 1	MINP1_HUMAN	X	X
Glypican-6	GPC6_HUMAN	X	X
IgGFc-binding protein	FCGBP_HUMAN	X	X
Immunoglobulin lambda variable 5-52	LV552_HUMAN	X	
Immunoglobulin kappa joining 1	KJ01_HUMAN	X	
Immunoglobulin kappa variable 3D-7	KVD07_HUMAN	X	
Endoribonuclease Dicer	DICER_HUMAN	X	
Dynamin-3	DYN3_HUMAN	X	
Receptor-interacting serine/threonine-protein kinase 3	RIPK3_HUMAN	X	
Transportin-3	TNPO3_HUMAN	X	
Lymphatic vessel endothelial hyaluronic acid receptor 1	LYVE1_HUMAN	X	

Cathepsin Z	CATZ_HUMAN	X	
PHD and RING finger domain-containing protein 1	PHRF1_HUMAN	X	
Dynein heavy chain 2, axonemal	DYH2_HUMAN	X	
Teneurin-3	TEN3_HUMAN	X	
Zinc finger C4H2 domain-containing protein	ZC4H2_HUMAN	X	
Dapper homolog 1	DACT1_HUMAN	X	
CASP8-associated protein 2	C8AP2_HUMAN	X	
Kinesin-like protein KIF13A	KI13A_HUMAN	X	
Band 4.1-like protein 4B	E41LB_HUMAN	X	
Threonine aspartase 1	TASP1_HUMAN	X	
Junctophilin-1	JPH1_HUMAN	X	
Leucine-rich repeat-containing protein 27	LRC27_HUMAN	X	
Succinyl-CoA:3-ketoacid coenzyme A transferase 2, mitochondrial	SCOT2_HUMAN	X	
Calcium-binding mitochondrial carrier protein SCaMC-3	SCMC3_HUMAN	X	
Matrix metalloproteinase-19	MMP19_HUMAN	X	
HIRA-interacting protein 3	HIRP3_HUMAN	X	
Cell division control protein 6 homolog	CDC6_HUMAN	X	
Zinc finger protein 184	ZN184_HUMAN	X	
RNA-binding protein 14	RBM14_HUMAN	X	
Hemicentin-1	HMCN1_HUMAN	X	
CDK5 regulatory subunit-associated protein 2	CK5P2_HUMAN	X	
T-cell immunoglobulin and mucin domain-containing protein 4	TIMD4_HUMAN	X	
Succinate--CoA ligase [GDP-forming] subunit beta, mitochondrial	SUCB2_HUMAN	X	
Putative transmembrane protein encoded by LINC00477	CL067_HUMAN	X	

Keratin, type I cuticular Ha5	KRT35_HUMAN	X	
Sodium-dependent phosphate transporter 1	S20A1_HUMAN	X	
InaD-like protein	INADL_HUMAN	X	
BPI fold-containing family B member 1	BPIB1_HUMAN	X	
FERM domain-containing protein 5	FRMD5_HUMAN	X	
Coiled-coil domain-containing protein 25	CCD25_HUMAN	X	
Cardiomyopathy-associated protein 5	CMYA5_HUMAN	X	
Leucine-rich repeat-containing protein 18	LRC18_HUMAN	X	
APC membrane recruitment protein 2	AMER2_HUMAN	X	
Myogenesis-regulating glycosidase	MYORG_HUMAN	X	
NIPA-like protein 3	NPAL3_HUMAN	X	
Plexin domain-containing protein 2	PXDC2_HUMAN	X	
Nephrocystin-3	NPHP3_HUMAN	X	
Dual specificity mitogen-activated protein kinase kinase 7	MP2K7_HUMAN	X	
ATP synthase mitochondrial F1 complex assembly factor 1	ATPF1_HUMAN	X	
Keratin, type II cytoskeletal 79	K2C79_HUMAN	X	
RNA-binding motif, single-stranded-interacting protein 2	RBMS2_HUMAN	X	
Nuclear receptor coactivator 1	NCOA1_HUMAN	X	
Laminin subunit alpha-4	LAMA4_HUMAN	X	
Nuclear mitotic apparatus protein 1	NUMA1_HUMAN	X	
Calicin	CALI_HUMAN	X	
Tubulin--tyrosine ligase-like protein 12	TTL12_HUMAN	X	
Metabotropic glutamate receptor 1	GRM1_HUMAN	X	
AP-3 complex subunit beta-2	AP3B2_HUMAN	X	
Mucin-2	MUC2_HUMAN	X	
Dystonin	DYST_HUMAN	X	

Prolyl endopeptidase FAP	SEPR_HUMAN	X	
Immunoglobulin lambda constant 6	IGLC6_HUMAN	X	
Immunoglobulin lambda constant 1	IGLC1_HUMAN	X	
Plasminogen-like protein A	PLGA_HUMAN	X	
Oxygen-regulated protein 1	RP1_HUMAN	X	
Putative macrophage stimulating 1-like protein	MST1L_HUMAN	X	
Tenascin-X	TENX_HUMAN	X	
ATP-binding cassette sub-family C member 8	ABCC8_HUMAN	X	
Keratin, type I cytoskeletal 17	K1C17_HUMAN	X	
Huntingtin	HD_HUMAN	X	
E-selectin	LYAM2_HUMAN	X	
Transcription factor jun-B	JUNB_HUMAN	X	
Collagen alpha-1(XVIII) chain	COIA1_HUMAN	X	
Collagen alpha-3(VI) chain	CO6A3_HUMAN	X	
Synaptobrevin homolog YKT6	YKT6_HUMAN	X	
Glutathione S-transferase theta-1	GSTT1_HUMAN	X	
Nuclear receptor corepressor 1	NCOR1_HUMAN	X	
Properdin	PROP_HUMAN		X
Immunoglobulin heavy variable 1-2	HV102_HUMAN		X
Phosphatidylcholine-sterol acyltransferase	LCAT_HUMAN		X
Cadherin-5	CADH5_HUMAN		X
Actin, cytoplasmic 2	ACTG_HUMAN		X
Transforming acidic coiled-coil-containing protein 3	TACC3_HUMAN		X
Immunoglobulin heavy variable 1-18	HV118_HUMAN		X
Immunoglobulin lambda-like polypeptide 1	IGLL1_HUMAN		X
Serum amyloid A-1 protein	SAA1_HUMAN		X
Lysine-specific demethylase 4C	KDM4C_HUMAN		X
Immunoglobulin lambda variable 6-57	LV657_HUMAN		X

Slit homolog 1 protein	SLIT1_HUMAN		X
Immunoglobulin kappa variable 1-16	KV116_HUMAN		X
Immunoglobulin kappa variable 6D-21	KVD21_HUMAN		X
Matrix metalloproteinase-9	MMP9_HUMAN		X
Coagulation factor XIII A chain	F13A_HUMAN		X
Immunoglobulin heavy variable 3-13	HV313_HUMAN		X
Basement membrane-specific heparan sulfate proteoglycan core protein	PGBM_HUMAN		X
Filamin-C	FLNC_HUMAN		X
Immunoglobulin kappa variable 1D-16	KVD16_HUMAN		X
Protein SOGA3	SOGA3_HUMAN		X
Pantetheinase	VNN1_HUMAN		X
Polymeric immunoglobulin receptor	PIGR_HUMAN		X
Platelet factor 4 variant	PF4V_HUMAN		X
Hepatocyte growth factor activator	HGFA_HUMAN		X
Villin-like protein	VILL_HUMAN		X
Mitochondrial import inner membrane translocase subunit Tim10 B	T10B_HUMAN		X
Carbonic anhydrase 2	CAH2_HUMAN		X
Immunoglobulin kappa variable 6-21	KV621_HUMAN		X
Beta-2-microglobulin	B2MG_HUMAN		X
Engulfment and cell motility protein 3	ELMO3_HUMAN		X
Immunoglobulin lambda variable 2-14	LV214_HUMAN		X
Immunoglobulin heavy variable 4-30-4	HVD34_HUMAN		X
E3 ubiquitin-protein ligase SHPRH	SHPRH_HUMAN		X
Vascular cell adhesion protein 1	VCAM1_HUMAN		X
Collectin-11	COL11_HUMAN		X

Immunoglobulin heavy variable 4-31	HV431_HUMAN		X
Tubulin polyglutamylase TTLL13P	TTL13_HUMAN		X
Trypsin-2	TRY2_HUMAN		X
Zinc finger protein 618	ZN618_HUMAN		X
N-acetylgalactosaminyltransferase 7	GALT7_HUMAN		X
Cell division cycle 5-like protein	CDC5L_HUMAN		X
Paired mesoderm homeobox protein 2B	PHX2B_HUMAN		X
Zinc finger protein 33A	ZN33A_HUMAN		X
Lactotransferrin	TRFL_HUMAN		X
Serine/threonine-protein kinase ATR	ATR_HUMAN		X
A disintegrin and metalloproteinase with thrombospondin motifs 9	ATS9_HUMAN		X
Immunoglobulin lambda variable 5-45	LV545_HUMAN		X
Immunoglobulin lambda variable 2-18	LV218_HUMAN		X
Keratin, type II cuticular Hb5	KRT85_HUMAN		X
Immunoglobulin heavy variable 3-33	HV333_HUMAN		X
Glycerol kinase 2	GLPK2_HUMAN		X
Calcium-independent phospholipase A2-gamma	PLPL8_HUMAN		X
Beta-ureidopropionase	BUP1_HUMAN		X
ARF GTPase-activating protein GIT1	GIT1_HUMAN		X
Transcription factor SOX-30	SOX30_HUMAN		X
Forkhead box protein O1	FOXO1_HUMAN		X
Complement factor H-related protein 3	FHR3_HUMAN		X
RasGAP-activating-like protein 1	RASL1_HUMAN		X
DENN domain-containing protein 5B	DEN5B_HUMAN		X
Signal-regulatory protein beta-1	SIRB1_HUMAN		X
Immunoglobulin lambda variable 7-46	LV746_HUMAN		X

Trypsin-3	TRY3_HUMAN		X
Transforming growth factor-beta-induced protein ig-h3	BGH3_HUMAN		X
Lysozyme C	LYSC_HUMAN		X
C-reactive protein	CRP_HUMAN		X
Filamin A-interacting protein 1-like	FIL1L_HUMAN		X
ADP-ribosyl cyclase/cyclic ADP-ribose hydrolase 2	BST1_HUMAN		X
Transcription factor AP-2-epsilon	AP2E_HUMAN		X
Exocyst complex component 7	EXOC7_HUMAN		X
Eukaryotic translation initiation factor 3 subunit C	EIF3C_HUMAN		X
Uncharacterized protein FLJ45252	YJ005_HUMAN		X
Titin	TITIN_HUMAN		X
Wee1-like protein kinase	WEE1_HUMAN		X
Beta-actin-like protein 2	ACTBL_HUMAN		X
Transforming acidic coiled-coil-containing protein 2	TACC2_HUMAN		X
Annexin A4	ANXA4_HUMAN		X
Putative methyltransferase NSUN6	NSUN6_HUMAN		X
Paraneoplastic antigen-like protein 8C	PNM8C_HUMAN		X
Cysteine-rich secretory protein 3	CRIS3_HUMAN		X
Neurofilament heavy polypeptide	NFH_HUMAN		X
Sorbin and SH3 domain-containing protein 1	SRBS1_HUMAN		X
Dermcidin	DCD_HUMAN		X
Keratin, type I cytoskeletal 15	K1C15_HUMAN		X
CD166 antigen	CD166_HUMAN		X
Adiponectin	ADIPO_HUMAN		X
Myeloperoxidase	PERM_HUMAN		X
Pleckstrin homology-like domain family A member	PHLA1_HUMAN		X

1			
ATP-binding cassette sub-family B member 5	ABCB5_HUMAN		X
L-lactate dehydrogenase A-like 6A	LDH6A_HUMAN		X
Kinesin-1 heavy chain	KINH_HUMAN		X
6-phosphogluconolactonase	6PGL_HUMAN		X
NEDD4-binding protein 1	N4BP1_HUMAN		X
cTAGE family member 9	CTGE9_HUMAN		X
Keratin, type II cytoskeletal 6C	K2C6C_HUMAN		X
Immunoglobulin heavy variable 7-4-1	HV741_HUMAN		X
Immunoglobulin heavy variable 1-69D	HV69D_HUMAN		X
LRP2-binding protein	LR2BP_HUMAN		X
Immunoglobulin heavy variable 4-30-2	HV432_HUMAN		X
Bromodomain and PHD finger-containing protein 3	BRPF3_HUMAN		X
Flavin reductase (NADPH)	BLVRB_HUMAN		X
Hemoglobin subunit delta	HBD_HUMAN		X
1-phosphatidylinositol 4,5-bisphosphate phosphodiesterase eta-1	PLCH1_HUMAN		X
Adenylate kinase isoenzyme 6	KAD6_HUMAN		X
Immunoglobulin heavy variable 3-53	HV353_HUMAN		X
Adenylate kinase isoenzyme 1	KAD1_HUMAN		X
Hemoglobin subunit gamma-1	HBG1_HUMAN		X
Receptor-type tyrosine-protein phosphatase gamma	PTPRG_HUMAN		X
Methanethiol oxidase	SBP1_HUMAN		X
Ribosomal protein S6 kinase alpha-3	KS6A3_HUMAN		X
Myosin-15	MYH15_HUMAN		X
Glutathione S-transferase omega-1	GSTO1_HUMAN		X
Copine-7	CPNE7_HUMAN		X

Importin subunit beta-1	IMB1_HUMAN		X
Glyceraldehyde-3-phosphate dehydrogenase	G3P_HUMAN		X
Rab GDP dissociation inhibitor beta	GDIB_HUMAN		X
Modulator of apoptosis 1	MOAP1_HUMAN		X
A-kinase anchor protein 9	AKAP9_HUMAN		X
Fibrous sheath-interacting protein 2	FSIP2_HUMAN		X
Immunoglobulin heavy variable 3-73	HV373_HUMAN		X
FYVE, RhoGEF and PH domain-containing protein 6	FGD6_HUMAN		X
Keratin, type I cuticular Ha1	K1H1_HUMAN		X
Lysine-specific demethylase 3B	KDM3B_HUMAN		X
Desmin	DESM_HUMAN		X
Coiled-coil domain-containing protein 28A	CC28A_HUMAN		X
Ephrin type-B receptor 3	EPHB3_HUMAN		X
Carboxypeptidase D	CBPD_HUMAN		X
Elongator complex protein 3	ELP3_HUMAN		X
Carbonic anhydrase 3	CAH3_HUMAN		X
Free fatty acid receptor 4	FFAR4_HUMAN		X
Coagulation factor VII	FA7_HUMAN		X
Protein ENL	ENL_HUMAN		X
Peroxiredoxin-2	PRDX2_HUMAN		X
Immunoglobulin kappa variable 1-8	KV108_HUMAN		X
Immunoglobulin lambda variable 3-9	LV39_HUMAN		X
Tensin-3	TENS3_HUMAN		X
Aminopeptidase N	AMPN_HUMAN		X
Intraflagellar transport protein 140 homolog	IF140_HUMAN		X
Nucleoprotein TPR	TPR_HUMAN		X

Syntaxin-binding protein 5-like	STB5L_HUMAN		X
Pulmonary surfactant-associated protein B	PSPB_HUMAN		X
SHC-transforming protein 3	SHC3_HUMAN		X
Tetratricopeptide repeat protein 7A	TTC7A_HUMAN		X
Matrix metalloproteinase-15	MMP15_HUMAN		X
TBC1 domain family member 1	TBCD1_HUMAN		X
Endothelial PAS domain-containing protein 1	EPAS1_HUMAN		X
GTP-binding protein 8	GTPB8_HUMAN		X
Immunoglobulin heavy variable 3-20	HV320_HUMAN		X
Cytoplasmic polyadenylation element-binding protein 4	CPEB4_HUMAN		X
Coiled-coil domain-containing protein 168	CC168_HUMAN		X
Dynein heavy chain 3, axonemal	DYH3_HUMAN		X
PR domain zinc finger protein 5	PRDM5_HUMAN		X
Protein S100-A9	S10A9_HUMAN		X
HHIP-like protein 2	HIPL2_HUMAN		X
Protein S100-A8	S10A8_HUMAN		X
Plastin-2	PLSL_HUMAN		X
Paraneoplastic antigen-like protein 6A	PNM6A_HUMAN		X
Cilia- and flagella-associated protein 100	CP100_HUMAN		X
DNA topoisomerase 1	TOP1_HUMAN		X
Putative trypsin-6	TRY6_HUMAN		X
Otogelin	OTOG_HUMAN		X
Arf-GAP with dual PH domain-containing protein 2	ADAP2_HUMAN		X
Sodium/hydrogen exchanger 1	SL9A1_HUMAN		X
Zinc finger protein 426	ZN426_HUMAN		X
MAP/microtubule affinity-regulating kinase 4	MARK4_HUMAN		X

Uncharacterized protein C6orf10	TSBP1_HUMAN		X
Histone-lysine N-methyltransferase SETD1A	SET1A_HUMAN		X
Macrophage metalloelastase	MMP12_HUMAN		X
WAP four-disulfide core domain protein 3	WFDC3_HUMAN		X
Spermatogenesis-associated protein 9	SPAT9_HUMAN		X
Protein ZGRF1	ZGRF1_HUMAN		X
Protein FAM110A	F110A_HUMAN		X
Inactive phospholipase D5	PLD5_HUMAN		X
Conserved oligomeric Golgi complex subunit 4	COG4_HUMAN		X
Receptor-type tyrosine-protein phosphatase delta	PTPRD_HUMAN		X
Zinc finger protein 404	ZN404_HUMAN		X
Structural maintenance of chromosomes protein 6	SMC6_HUMAN		X
E3 ubiquitin-protein ligase TRIM7	TRIM7_HUMAN		X
Immunoglobulin heavy variable 4-39	HV439_HUMAN		X
Transcription factor SPT20 homolog	SP20H_HUMAN		X
Inactive histone-lysine N-methyltransferase 2E	KMT2E_HUMAN		X
Phosphopantothenate--cysteine ligase	PPCS_HUMAN		X
Unconventional myosin-XV	MYO15_HUMAN		X
Glutaredoxin domain-containing cysteine-rich protein 2	GRCR2_HUMAN		X
Sorting nexin-25	SNX25_HUMAN		X
Zinc finger protein 622	ZN622_HUMAN		X
Ankyrin repeat and SOCS box protein 7	ASB7_HUMAN		X
Kinesin heavy chain isoform 5A	KIF5A_HUMAN		X
Immunoglobulin heavy variable 3-21	HV321_HUMAN		X
Fibrinogen beta chain	FIBB_HUMAN		X
Probable guanine nucleotide exchange factor	MF2L2_HUMAN		X

MCF2L2			
Coiled-coil domain-containing protein 106	CC106_HUMAN		X
Neurexin-3	NRX3A_HUMAN		X
Interferon regulatory factor 7	IRF7_HUMAN		X

Table S3. Proteins identified in the protein corona formed after the interaction of AuNPs (10.02 ± 0.91 nm) (30 min, incubation) with serum samples belonging to breast cancer patients with different subtypes: luminal A (n = 11), luminal B HER2 negative (n = 10), luminal B HER2 positive (n = 7), HER2 positive (n = 6), and triple negative (n=8). The accession number, gene name and species (Human) were reported.

Protein Name	UniProt Name	Entry Name	Gene	Luminal A	Luminal B HER2 negative	Luminal B HER2 positive	HER2 positive	Triple negative
Properdin	PROP_HUMAN	P27918	CFP	X	X	X	X	X
Immunoglobulin heavy variable 1-2	HV102_HUMAN	P23083	IGHV1-2	X	X	X	X	X
Phosphatidylcholine-sterol acyltransferase	LCAT_HUMAN	P04180	LCAT	X	X	X	X	X
Cadherin-5	CADH5_HUMAN	P33151	CDH5	X	X	X	X	X
Actin, cytoplasmic 2	ACTG_HUMAN	P63261	ACTG1	X	X	X	X	X
Transforming acidic coiled-coil-containing protein 3	TACC3_HUMAN	Q9Y6A5	TACC3	X	X	X	X	
Immunoglobulin heavy variable 1-18	HV118_HUMAN	A0A0C4 DH31	IGHV1-18	X	X	X	X	
Immunoglobulin lambda-like polypeptide 1	IGLL1_HUMAN	P15814	IGLL1	X	X	X	X	
Serum amyloid A-1 protein	SAA1_HUMAN	P0DJI8	SAA1	X	X	X		X
Lysine-specific demethylase 4C	KDM4C_HUMAN	Q9H3R0	KDM4C	X	X	X		X
Immunoglobulin lambda variable 6-57	LV657_HUMAN	P01721	IGLV6-57	X	X	X		X
Slit homolog 1 protein	SLIT1_HUMAN	O75093	SLIT1	X	X	X		X
Immunoglobulin kappa variable 1-16	KV116_HUMAN	P04430	IGKV1-16	X	X	X		X
Immunoglobulin kappa variable 6D-21	KVD21_HUMAN	A0A0A0 MT36	IGKV6D-21	X	X	X		X
Matrix metalloproteinase-9	MMP9_HUMAN	P14780	MMP9	X		X	X	X

Coagulation factor XIII A chain	F13A_HUMAN	P00488	F13A1	X		X	X	X
Immunoglobulin heavy variable 3-13	HV313_HUMAN	P01766	IGHV3-13	X	X	X		
Basement membrane-specific heparan sulfate proteoglycan core protein	PGBM_HUMAN	P98160	HSPG2	X	X	X		
Filamin-C	FLNC_HUMAN	Q14315	FLNC	X	X			X
Immunoglobulin kappa variable 1D-16	KVD16_HUMAN	P01601	IGKV1D-16	X	X			X
Protein SOGA3	SOGA3_HUMAN	Q5TF21	SOGA3	X	X			X
Pantetheinase	VNN1_HUMAN	O95497	VNN1	X	X			X
Polymeric immunoglobulin receptor	PIGR_HUMAN	P01833	PIGR	X		X		X
Platelet factor 4 variant	PF4V_HUMAN	P10720	PF4V1	X		X		X
Hepatocyte growth factor activator	HGFA_HUMAN	Q04756	HGFAC	X			X	X
Villin-like protein	VILL_HUMAN	O15195	VILL		X	X	X	
Mitochondrial import inner membrane translocase subunit Tim10 B	T10B_HUMAN	Q9Y5J6	TIMM10 B		X	X	X	
Carbonic anhydrase 2	CAH2_HUMAN	P00918	CA2		X	X		X
Immunoglobulin kappa variable 6-21	KV621_HUMAN	A0A0C4 DH24	IGKV6-21		X		X	X
Beta-2-microglobulin	B2MG_HUMAN	P61769	B2M	X	X			
Engulfment and cell motility protein 3	ELMO3_HUMAN	Q96BJ8	ELMO3	X	X			
Immunoglobulin lambda variable 2-14	LV214_HUMAN	P01704	IGLV2-14	X	X			
Immunoglobulin heavy variable 4-30-4	HVD34_HUMAN	P0DP06	IGHV4-30-4	X	X			
E3 ubiquitin-protein ligase SHPRH	SHPRH_HUMAN	Q149N8	SHPRH	X	X			
Vascular cell adhesion protein 1	VCAM1_HUMAN	P19320	VCAM1	X	X			

Collectin-11	COL11_HUMAN	Q9BWP8	COLEC1 1	X	X			
Immunoglobulin heavy variable 4-31	HV431_HUMAN	P0DP07	IGHV4-31	X	X			
Tubulin polyglutamylase TTLL13P	TTL13_HUMAN	A6NNM8	TTLL13P	X		X		
Trypsin-2	TRY2_HUMAN	P07478	PRSS2	X		X		
Zinc finger protein 618	ZN618_HUMAN	Q5T7W0	ZNF618	X		X		
N-acetylgalactosaminyltransferase 7	GALT7_HUMAN	Q86SF2	GALNT7	X		X		
Cell division cycle 5-like protein	CDC5L_HUMAN	Q99459	CDC5L	X			X	
Paired mesoderm homeobox protein 2B	PHX2B_HUMAN	Q99453	PHOX2B	X			X	
Zinc finger protein 33A	ZN33A_HUMAN	Q06730	ZNF33A	X				X
Lactotransferrin	TRFL_HUMAN	P02788	LTF	X				X
Serine/threonine-protein kinase ATR	ATR_HUMAN	Q13535	ATR	X				X
A disintegrin and metalloproteinase with thrombospondin motifs 9	ATS9_HUMAN	Q9P2N4	ADAMT S9	X				X
Immunoglobulin lambda variable 5-45	LV545_HUMAN	A0A087 WSX0	IGLV5-45		X	X		
Immunoglobulin lambda variable 2-18	LV218_HUMAN	A0A075B 6J9	IGLV2-18		X	X		
Keratin, type II cuticular Hb5	KRT85_HUMAN	P78386	KRT85		X	X		
Immunoglobulin heavy variable 3-33	HV333_HUMAN	P01772	IGHV3-33		X	X		
Glycerol kinase 2	GLPK2_HUMAN	Q14410	GK2		X		X	
Calcium-independent phospholipase A2-gamma	PLPL8_HUMAN	Q9NP80	PNPLA8		X			X
Beta-ureidopropionase	BUP1_HUMAN	Q9UBR1	UPB1		X			X
ARF GTPase-activating protein GIT1	GIT1_HUMAN	Q9Y2X7	GIT1		X			X
Transcription factor SOX-30	SOX30_HUMAN	O94993	SOX30		X			X

Forkhead box protein O1	FOXO1_HUMAN	Q12778	FOXO1			X	X	
Complement factor H-related protein 3	FHR3_HUMAN	Q02985	CFHR3			X	X	
RasGAP-activating-like protein 1	RASL1_HUMAN	O95294	RASAL1			X		X
DENN domain-containing protein 5B	DEN5B_HUMAN	Q6ZUT9	DENND5B			X		X
Signal-regulatory protein beta-1	SIRB1_HUMAN	O00241	SIRPB1			X		X
Immunoglobulin lambda variable 7-46	LV746_HUMAN	A0A075B6I9	IGLV7-46			X		X
Trypsin-3	TRY3_HUMAN	P35030	PRSS3				X	X
Transforming growth factor-beta-induced protein ig-h3	BGH3_HUMAN	Q15582	TGFBI				X	X
Lysozyme C	LYSC_HUMAN	P61626	LYZ	X				
C-reactive protein	CRP_HUMAN	P02741	CRP	X				
Filamin A-interacting protein 1-like	FIL1L_HUMAN	Q4L180	FILIP1L	X				
ADP-ribosyl cyclase/cyclic ADP-ribose hydrolase 2	BST1_HUMAN	Q10588	BST1	X				
Transcription factor AP-2-epsilon	AP2E_HUMAN	Q6VUC0	TFAP2E	X				
Exocyst complex component 7	EXOC7_HUMAN	Q9UPT5	EXOC7	X				
Eukaryotic translation initiation factor 3 subunit C	EIF3C_HUMAN	Q99613	EIF3C	X				
Uncharacterized protein FLJ45252	YJ005_HUMAN	Q6ZSR9	N/A	X				
Titin	TITIN_HUMAN	Q8WZ42	TTN	X				
Wee1-like protein kinase	WEE1_HUMAN	P30291	WEE1	X				
Beta-actin-like protein 2	ACTBL_HUMAN	Q562R1	ACTBL2	X				
Transforming acidic coiled-coil-containing protein 2	TACC2_HUMAN	O95359	TACC2	X				
Annexin A4	ANXA4_HUMAN	P09525	ANXA4	X				
Putative methyltransferase NSUN6	NSUN6_HUMAN	Q8TEA1	NSUN6	X				

Paraneoplastic antigen-like protein 8C	PNM8C_HUMAN	A0A1B0GUJ8	PNMA8C	X				
Cysteine-rich secretory protein 3	CRIS3_HUMAN	P54108	CRISP3	X				
Neurofilament heavy polypeptide	NFH_HUMAN	P12036	NEFH	X				
Sorbin and SH3 domain-containing protein 1	SRBS1_HUMAN	Q9BX66	SORBS1	X				
Dermcidin	DCD_HUMAN	P81605	DCD	X				
Keratin, type I cytoskeletal 15	K1C15_HUMAN	P19012	KRT15	X				
CD166 antigen	CD166_HUMAN	Q13740	ALCAM	X				
Adiponectin	ADIPO_HUMAN	Q15848	ADIPOQ	X				
Myeloperoxidase	PERM_HUMAN	P05164	MPO	X				
Pleckstrin homology-like domain family A member 1	PHLA1_HUMAN	Q8WV24	PHLDA1	X				
ATP-binding cassette sub-family B member 5	ABCB5_HUMAN	Q2M3G0	ABCB5	X				
L-lactate dehydrogenase A-like 6A	LDH6A_HUMAN	Q6ZMR3	LDHAL6A	X				
Kinesin-1 heavy chain	KINH_HUMAN	P33176	KIF5B	X				
6-phosphogluconolactonase	6PGL_HUMAN	O95336	PGLS	X				
NEDD4-binding protein 1	N4BP1_HUMAN	O75113	N4BP1	X				
cTAGE family member 9	CTGE9_HUMAN	A4FU28	CTAGE9		X			
Keratin, type II cytoskeletal 6C	K2C6C_HUMAN	P48668	KRT6C		X			
Immunoglobulin heavy variable 7-4-1	HV741_HUMAN	A0A0J9YVY3	IGHV7-4-1		X			
Immunoglobulin heavy variable 1-69D	HV69D_HUMAN	A0A0B4J2H0	IGHV1-69D		X			
LRP2-binding protein	LR2BP_HUMAN	Q9P2M1	LRP2BP		X			
Immunoglobulin heavy variable 4-30-2	HV432_HUMAN	A0A087WSY4	IGHV4-30-2		X			
Bromodomain and PHD finger-	BRPF3_HUMAN	Q9ULD4	BRPF3		X			

containing protein 3								
Flavin reductase (NADPH)	BLVRB_HUMAN	P30043	BLVRB		X			
Hemoglobin subunit delta	HBD_HUMAN	P02042	HBD		X			
1-phosphatidylinositol 4,5-bisphosphate phosphodiesterase eta-1	PLCH1_HUMAN	Q4KWH8	PLCH1		X			
Adenylate kinase isoenzyme 6	KAD6_HUMAN	Q9Y3D8	AK6		X			
Immunoglobulin heavy variable 3-53	HV353_HUMAN	P01767	IGHV3-53		X			
Adenylate kinase isoenzyme 1	KAD1_HUMAN	P00568	AK1		X			
Hemoglobin subunit gamma-1	HBG1_HUMAN	P69891	HBG1		X			
Receptor-type tyrosine-protein phosphatase gamma	PTPRG_HUMAN	P23470	PTPRG		X			
Methanethiol oxidase	SBP1_HUMAN	Q13228	SELENBP1		X			
Ribosomal protein S6 kinase alpha-3	KS6A3_HUMAN	P51812	RPS6KA3		X			
Myosin-15	MYH15_HUMAN	Q9Y2K3	MYH15		X			
Glutathione S-transferase omega-1	GSTO1_HUMAN	P78417	GSTO1		X			
Copine-7	CPNE7_HUMAN	Q9UBL6	CPNE7		X			
Importin subunit beta-1	IMB1_HUMAN	Q14974	KPNB1		X			
Glyceraldehyde-3-phosphate dehydrogenase	G3P_HUMAN	P04406	GAPDH		X			
Rab GDP dissociation inhibitor beta	GDIB_HUMAN	P50395	GDI2		X			
Modulator of apoptosis 1	MOAP1_HUMAN	Q96BY2	MOAP1		X			
A-kinase anchor protein 9	AKAP9_HUMAN	Q99996	AKAP9		X			
Fibrous sheath-interacting protein 2	FSIP2_HUMAN	Q5CZC0	FSIP2		X			
Immunoglobulin heavy variable 3-73	HV373_HUMAN	A0A0B4J1V6	IGHV3-73		X			
FYVE, RhoGEF and PH domain-containing protein 6	FGD6_HUMAN	Q6ZV73	FGD6		X			

Keratin, type I cuticular Ha1	K1H1_HUMAN	Q15323	KRT31		X			
Lysine-specific demethylase 3B	KDM3B_HUMAN	Q7LBC6	KDM3B		X			
Desmin	DESM_HUMAN	P17661	DES		X			
Coiled-coil domain-containing protein 28A	CC28A_HUMAN	Q8IWP9	CCDC28 A		X			
Ephrin type-B receptor 3	EPHB3_HUMAN	P54753	EPHB3		X			
Carboxypeptidase D	CBPD_HUMAN	O75976	CPD		X			
Elongator complex protein 3	ELP3_HUMAN	Q9H9T3	ELP3		X			
Carbonic anhydrase 3	CAH3_HUMAN	P07451	CA3		X			
Free fatty acid receptor 4	FFAR4_HUMAN	Q5NUL3	FFAR4		X			
Coagulation factor VII	FA7_HUMAN	P08709	F7		X			
Protein ENL	ENL_HUMAN	Q03111	MLLT1		X			
Peroxiredoxin-2	PRDX2_HUMAN	P32119	PRDX2		X			
Immunoglobulin kappa variable 1-8	KV108_HUMAN	A0A0C4 DH67	IGKV1-8		X			
Immunoglobulin lambda variable 3-9	LV39_HUMAN	A0A075B 6K5	IGLV3-9			X		
Tensin-3	TENS3_HUMAN	Q68CZ2	TNS3			X		
Aminopeptidase N	AMPN_HUMAN	P15144	ANPEP			X		
Intraflagellar transport protein 140 homolog	IF140_HUMAN	Q96RY7	IFT140			X		
Nucleoprotein TPR	TPR_HUMAN	P12270	TPR			X		
Syntaxin-binding protein 5-like	STB5L_HUMAN	Q9Y2K9	STXBP5 L			X		
Pulmonary surfactant-associated protein B	PSPB_HUMAN	P07988	SFTPB			X		
SHC-transforming protein 3	SHC3_HUMAN	Q92529	SHC3			X		
Tetratricopeptide repeat protein 7A	TTC7A_HUMAN	Q9ULT0	TTC7A			X		
Matrix metalloproteinase-15	MMP15_HUMAN	P51511	MMP15			X		

TBC1 domain family member 1	TBCD1_HUMAN	Q86TI0	TBC1D1			X		
Endothelial PAS domain-containing protein 1	EPAS1_HUMAN	Q99814	EPAS1			X		
GTP-binding protein 8	GTPB8_HUMAN	Q8N3Z3	GTPBP8			X		
Immunoglobulin heavy variable 3-20	HV320_HUMAN	A0A0C4 DH32	IGHV3-20			X		
Cytoplasmic polyadenylation element-binding protein 4	CPEB4_HUMAN	Q17RY0	CPEB4			X		
Coiled-coil domain-containing protein 168	CC168_HUMAN	Q8NDH2	CCDC168			X		
Dynein heavy chain 3, axonemal	DYH3_HUMAN	Q8TD57	DNAH3			X		
PR domain zinc finger protein 5	PRDM5_HUMAN	Q9NQX1	PRDM5			X		
Protein S100-A9	S10A9_HUMAN	P06702	S100A9			X		
HHIP-like protein 2	HIPL2_HUMAN	Q6UWX4	HHIPL2			X		
Protein S100-A8	S10A8_HUMAN	P05109	S100A8			X		
Plastin-2	PLSL_HUMAN	P13796	LCP1			X		
Paraneoplastic antigen-like protein 6A	PNM6A_HUMAN	P0CW24	PNMA6A				X	
Cilia- and flagella-associated protein 100	CP100_HUMAN	Q494V2	CFAP100				X	
DNA topoisomerase 1	TOP1_HUMAN	P11387	TOP1				X	
Putative trypsin-6	TRY6_HUMAN	Q8NHM4	PRSS3P2				X	
Otogelin	OTOG_HUMAN	Q6ZRI0	OTOG				X	
Arf-GAP with dual PH domain-containing protein 2	ADAP2_HUMAN	Q9NPF8	ADAP2				X	
Sodium/hydrogen exchanger 1	SL9A1_HUMAN	P19634	SLC9A1				X	
Zinc finger protein 426	ZN426_HUMAN	Q9BUY5	ZNF426				X	
MAP/microtubule affinity-regulating kinase 4	MARK4_HUMAN	Q96L34	MARK4				X	
Uncharacterized protein C6orf10	TSBP1_HUMAN	Q5SRN2	TSBP1				X	

Histone-lysine N-methyltransferase SETD1A	SET1A_HUMAN	O15047	SETD1A				X	
Macrophage metalloelastase	MMP12_HUMAN	P39900	MMP12				X	
WAP four-disulfide core domain protein 3	WFDC3_HUMAN	Q8IUB2	WFDC3				X	
Spermatogenesis-associated protein 9	SPAT9_HUMAN	Q9BWV2	SPATA9				X	
Protein ZGRF1	ZGRF1_HUMAN	Q86YA3	ZGRF1				X	
Protein FAM110A	F110A_HUMAN	Q9BQ89	FAM110 A					X
Inactive phospholipase D5	PLD5_HUMAN	Q8N7P1	PLD5					X
Conserved oligomeric Golgi complex subunit 4	COG4_HUMAN	Q9H9E3	COG4					X
Receptor-type tyrosine-protein phosphatase delta	PTPRD_HUMAN	P23468	PTPRD					X
Zinc finger protein 404	ZN404_HUMAN	Q494X3	ZNF404					X
Structural maintenance of chromosomes protein 6	SMC6_HUMAN	Q96SB8	SMC6					X
E3 ubiquitin-protein ligase TRIM7	TRIM7_HUMAN	Q9C029	TRIM7					X
Immunoglobulin heavy variable 4-39	HV439_HUMAN	P01824	IGHV4-39					X
Transcription factor SPT20 homolog	SP20H_HUMAN	Q8NEM7	SUPT20H					X
Inactive histone-lysine N-methyltransferase 2E	KMT2E_HUMAN	Q8IZD2	KMT2E					X
Phosphopantothenate--cysteine ligase	PPCS_HUMAN	Q9HAB8	PPCS					X
Unconventional myosin-XV	MYO15_HUMAN	Q9UKN7	MYO15A					X
Glutaredoxin domain-containing cysteine-rich protein 2	GRCR2_HUMAN	A6NFK2	GRXCR2					X
Sorting nexin-25	SNX25_HUMAN	Q9H3E2	SNX25					X
Zinc finger protein 622	ZN622_HUMAN	Q969S3	ZNF622					X
Ankyrin repeat and SOCS box	ASB7_HUMAN	Q9H672	ASB7					X

protein 7								
Kinesin heavy chain isoform 5A	KIF5A_HUMAN	Q12840	KIF5A					X
Immunoglobulin heavy variable 3-21	HV321_HUMAN	A0A0B4J 1V1	IGHV3-21					X
Fibrinogen beta chain	FIBB_HUMAN	P02675	FGB					X
Probable guanine nucleotide exchange factor MCF2L2	MF2L2_HUMAN	Q86YR7	MCF2L2					X
Coiled-coil domain-containing protein 106	CC106_HUMAN	Q9BWC9	CCDC10 6					X
Neurexin-3	NRX3A_HUMAN	Q9Y4C0	NRXN3					X
Interferon regulatory factor 7	IRF7_HUMAN	Q92985	IRF7					X
